# An assessment of latrine front-end characteristics and associated surface *E. coli* indicated faecal contamination in rural Fiji

**DOI:** 10.1007/s11356-024-34668-x

**Published:** 2024-08-21

**Authors:** Sabita Adhikari, Shylett Anthony, Ponipate Baleinamau, Jeremaia Coriakula, Thompson Daurewa, Rachel Devi, Sikeli Gavidi, Pierre Horwitz, Erin C. Hunter, Aaron Jenkins, Stacy Jupiter, Maria Lalamacuata, Kinikoto Mailautoka, Sangeeta Mangubhai, Kelera Naivalu, Timoci Naivalulevu, Vilisi Naivalulevu, Nabeela Nasim, Sikeli Naucunivanua, Joel Negin, Paul van Nimwegen, Anaseini Ratu, Mereia Ravoka, Andrew Tukana, Jack van de Vossenberg, Donald Wilson, Jacqueline Thomas

**Affiliations:** 1 School of Civil Engineering, The University of Sydney, Darlington, NSW 2006 Australia; 2 Fiji Institute of Pacific Health Research, College of Medicine, Nursing & Health Sciences, Fiji National University, Hoodless House, Suva, Fiji; 3 Centre for People, Place, and Planet, Edith Cowan University, Joondalup, WA Australia; 4 Department of Public Health Sciences, College of Behavioural, Social and Health Sciences, Clemson University, Clemson, USA; 5 School of Public Health, Faculty of Medicine and Health, The University of Sydney, Camperdown, NSW 2006 Australia; 6 Wildlife Conservation Society, Melanesia Program, Suva, Fiji; 7 Wildlife Conservation Society, Fiji Program, Suva, Fiji; 8 Talanoa Consulting, 42 Knollys Street, Suva, Fiji; 9 Water Supply, Sanitation and Environmental Engineering Department, IHE Delft Institute of Water Education, Delft, The Netherlands

**Keywords:** Faecal pathogen transmission pathways, Frequent human contact surfaces, Latrine surfaces, Latrine usage behaviour, Microbial risks, Pacific Islands, Sustainable Development Goal 6

## Abstract

In Fiji, 90% of the population has access to basic sanitation; however, there are still persistent health risks from endemic faecal-oral diseases such as typhoid fever. There is a need to assess the contribution of existing sanitation facilities in the faecal pathogen transmission pathway. This study was conducted as part of a larger planetary health study across 29 rural communities within five river catchments. This specific research aimed to characterise latrine front-ends, both infrastructure and usage behaviour, and to assess the faecal contamination levels on various frequently contacted latrine surfaces in rural Fiji. A sanitation survey, along with observation and latrine swab sampling, was conducted in households over three phases: baseline (*n* = 311) (Aug–Dec 2019), endline (*n* = 262) (Jun–Sep 2022) and an in-depth front-end study (*n* = 12) (Oct–Nov 2022). Of 311 households, almost all had pedestal-type latrines, predominately cistern-flush (83%), followed by pour-flush (13%), and then hole-type (pit) latrines (4%). Washable latrine floors had significantly higher *E. coli* densities (6.7 × 10^2^ CFU/25 cm^2^) compared to non-washable floors (1.3 × 10^2^ CFU/25 cm^2^) (*p* = 0.05), despite washable floors indicating improved latrines. The in-depth front-end analysis found that moist latrine surfaces had significantly elevated *E. coli* densities (1.2 × 10^3^ CFU/25 cm^2^) compared to the dry ones (14.3 CFU/25 cm^2^) (*p* < 0.001), highlighting the importance of maintaining dry latrine surfaces. Latrine floors and mid-walls were the most frequently contaminated surfaces, emphasising the need to clean and disinfect these surfaces. Only 46% of the households reported always using soap for handwashing after defecation, exacerbating the risk of transmitting faecal pathogens. This study highlights that latrine cleanliness and hygiene are as crucial as latrine infrastructures for the effective disruption of faecal pathogens transmission during latrine use.

**Supplementary Information:**

The online version contains supplementary material available at 10.1007/s11356-024-34668-x.

## Introduction

Inadequate sanitation remains a global health challenge contributing to the transmission of several infectious diseases, accounting for an estimated 432,000 deaths annually (WHO 2022). While most sanitation-related diseases are transmitted through faecal-oral pathways (such as typhoid), some can be transmitted via skin contact with faecally contaminated soil (such as hookworm) and poor hygiene (such as trachoma) (Hutton and Chase 2016; WHO 2022). The microbial pathogens causing these diseases spread through various pathways including water, flies, soil, hands, surfaces and contaminated food (Curtis et al. 2000; Navab-Daneshmand et al. 2018). Improving sanitation infrastructures and hygiene practices remains crucial to break these transmission pathways and reduce microbial risks (Adhikari et al. 2023).

The WHO/UNICEF Joint Monitoring Program (JMP) defines the sanitation ladder with five service levels ranging from open defecation at the bottom to unimproved, limited, basic and safely managed sanitation (WHO and UNICEF 2021). Achieving basic sanitation involves using improved sanitation facilities, such as flush or pour-flush latrines and pit latrines with slabs, that hygienically separate the users from faeces and are not shared with other households. Safely managed sanitation is defined as using private improved sanitation facilities where human excreta is treated safely onsite or safely transported and treated offsite, which is the key indicator of progress for the Sustainable Development Goal (SDG) 6. While significant progress has been made globally in achieving access to improved sanitation facilities since the Millennium Development Goals (Weststrate et al. 2019; WHO 2015), the persistence of sanitation-related diseases underscores that access to improved sanitation infrastructure alone cannot effectively break pathogen transmission (Behera et al. 2021; Odagiri et al. 2016). Therefore, it is essential to assess the safe use of latrines and hygiene practices such as handwashing with soap alongside efforts to expand sanitation coverage (Behera et al. 2021; Dey et al. 2019).

Onsite sanitation systems (non-sewered sanitation), such as pit latrines with slabs and septic systems, are the primary form of improved sanitation in rural and peri-urban communities (Gwenzi et al. 2023; Twinomucunguzi et al. 2020; WHO and UNICEF 2017). Latrine front-ends or user interface is the first step of the sanitation service chain which includes options such as pedestals, squat plates, or holes in the ground, with flush systems depending on water availability (Thomas and Gold 2020). Non-technical factors such as latrine usage behaviours and socio-cultural aspects can influence the extent of microbial risks and transmission from latrines (Mahdavinejad et al. 2011; Stenström et al. 2011). Frequent interactions between latrine front-ends and users create an ideal environment for pathogen transmission via contaminated hands and surfaces leading to skin, gastrointestinal or respiratory infections among household members (Abney et al. 2021; Bloomfield et al. 2017, 2012).

While the risk of pathogen transmission from contaminated surfaces including latrine surfaces is long known, the recent COVID-19 pandemic brought this to significant attention, highlighting the need to study contaminated latrine surfaces (Sharma et al. 2023; Sivamuni et al. 2022), particularly given the lack of field data on latrine front-end infrastructures and usage behaviours in the sanitation literature (Adhikari et al. 2023). The knowledge gap is even more pronounced in Pacific Island countries where there are variations in latrine front-end types. Cistern flush latrines are predominantly reported in countries such as Fiji (Nasim et al. 2023) and Tonga (White et al. 2020), whereas hole-type (without water seal) latrines are commonly reported in Papua New Guinea (Seidahmed et al. 2021). Furthermore, only ten studies globally reported field measurements of microbial densities on various front-end surfaces of household latrines; with none from Pacific Island countries (Adhikari et al. 2023). A thorough assessment of latrine front-end characteristics and usage behaviours in the Pacific is thus warranted to assess microbial risks and potential contamination of the latrine surfaces.

Fiji is well positioned for an in-depth investigation given the high coverage of improved sanitation coupled with the high incidence of faecal-oral diseases. Recent JMP statistics report a 93% overall coverage for improved sanitation facilities that are not shared in Fiji, which includes access to basic (44%) and safely managed sanitation service levels (49%) (WHO and UNICEF 2022). This coverage of improved sanitation facilities in Fiji is evenly distributed between urban and rural areas, with both reporting a consistent 93% coverage rate. Fiji still has a significant burden of faecal-oral diseases such as typhoid fever, with inadequate sanitation identified as one of the risk factors (Prasad et al. 2018; Watson et al. 2017). Previous studies assessing the risk factors of faecal-oral diseases in Fiji commonly highlight the transmission routes, including consumption of contaminated surface or groundwater and unwashed produce exposed to contamination due to leaching or flooding through unsafe latrine back-ends (Jenkins et al. 2016, 2019; Prasad et al. 2018; Thompson et al. 2014). However, there is limited information on latrine front-end infrastructures and usage behaviours from Fiji, and no studies have measured the faecal contamination levels on latrine front-end surfaces. Further, the association between latrine front-end types and usage behaviours with the faecal contamination levels on latrine surfaces has not yet been explored.

This study aims to address these gaps by assessing faecal contamination levels (*Escherichia coli* densities) on latrine front-end surfaces across 29 rural communities in Fiji. The specific objectives are to (a) investigate variability in the existing latrine front-end types and associated usage behaviours; (b) identify the latrine front-end types with the highest faecal contamination levels on latrine floors; (c) assess the faecal contamination levels on frequent human contact surfaces within latrine front-end; and (d) identify the factors that impact the faecal contamination levels on these identified surfaces.

## Materials and methods

### Study location

The latrine front-end analysis presented in this study is part of a broader programme of work under the Watershed Interventions for Systems Health in Fiji (WISH Fiji) project designed specifically to identify and address multiple drivers of negative health impacts on people and the environment that operate and interact at nested scales within watersheds (Jupiter et al. 2024). Fiji has an estimated population of 930,000 (The World Bank 2022) and has been classified as a high human development index (HDI) country (UNDP 2022). Fiji consists of over 330 islands, with the majority of the population located on the two major islands: Viti Levu and Vanua Levu. The WISH Fiji project covered five river catchments: Dawasamu, Upper Navua and Waibula (located on Viti Levu), Dama (located on Vanua Levu) and Bureta (located on Ovalau Island). A total of 29 communities were selected from these catchments, which included seven from Bureta, six from Dama, five from Dawasamu, five from Upper Navua and six from Waibula. The selection of these communities was primarily based on their geographical positioning along the existing major rivers and the prevalence of faecal-oral diseases such as typhoid fever, as previously described by Jupiter et al. 2024.

The Natadradave community in the Dawasamu catchment was selected to conduct an in-depth front-end analysis of the faecal contamination levels on the contact surfaces of latrines. Natadradave is situated around 60 km north of the capital, Suva. It was selected for the front-end sampling due to its proximity to the laboratory in Suva and the availability of a wide range of latrine front-end types compared to other communities in Dawasamu.

#### Sanitation and household survey

**Fig. 1:**
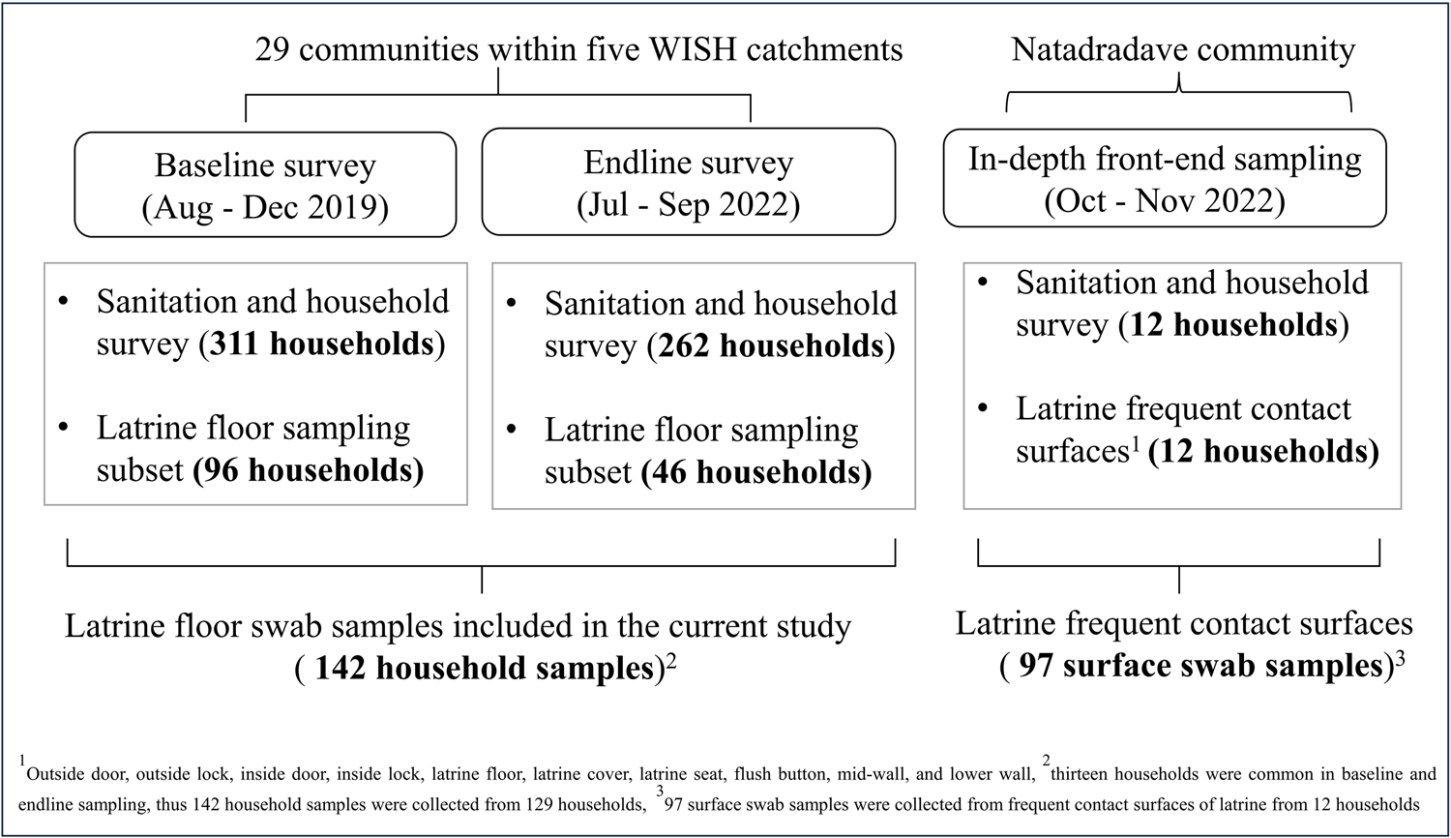
Overview of latrine swab collection process for assessing faecal contamination levels on latrine front-end surfaces in rural Fiji included in this study

Sanitation surveys, observations and latrine swab sampling were carried out in three phases: baseline (Aug–Dec 2019), endline (Jun–Sep 2022) and in-depth front-end study (Oct–Nov 2022) (Fig. 1). For baseline and endline, household surveys and infrastructure observations were carried out by trained enumerators covering 311 households in baseline and 262 households in endline. The same households surveyed from baseline were revisited for endline but with some cases of no one being home, leading to lower responses in the endline survey. This random sample of households represented 21% and 17% of the total households in the 29 selected communities (1502 households), respectively. An adult member of each household was interviewed using structured questionnaires that covered socio-economic status, demographics of latrine users, usage behaviours such as anal cleansing methods and handwashing and reported diseases. Further, latrines were visually inspected by the enumerators using sanitation observation checklists for the type of latrine front-ends, latrine floors, presence of anal cleansing materials and handwashing facilities. GPS coordinates and photos of the latrine front and back-ends were also captured. In addition, community-level sanitation information was also collected through the process of sanitation safety planning (SSP) from 2020 to 2021 (Nasim et al. 2023). The SSP approach involved the engagement and active participation of community leaders, community health workers, water safety committees and key residents to gather the required community-wide sanitation data. It also included educating these community leaders on sanitation infrastructures and their maintenance. For the in-depth front-end study, more detailed sanitation surveys and observations were carried out in 12 households. The survey questionnaires and observation checklists were adapted from baseline and endline surveys, including additional questions focused on the characteristics and maintenance of the latrine front-ends and usage behaviours, such as frequency of cleaning latrines, materials used for cleaning and menstrual hygiene management practices (Table S1). Visual assessment was also conducted to record the presence of moisture, dirt, material type and texture for each sampling surface of latrines.

#### Latrine swab sample collection

The latrine swab samples were collected in three phases following the same timeline as sanitation and household surveys. Latrine floor swab samples were collected from a random selection of 96 out of 311 households in baseline and 46 out of 262 households in endline (Fig. 1). Sterile dry cotton swabs with wooden handles (Puritan®, USA) were used to collect the samples from an approximately 5 cm × 5 cm area for around 30 s on the latrine floor around the pedestal or squat plate where a user would place their feet. The swabs were collected in labelled 15-ml falcon tubes (Biologix®, USA), cold stored in cool boxes and transported back to the laboratory for processing and analysis within 24 h.

**Fig. 2:**
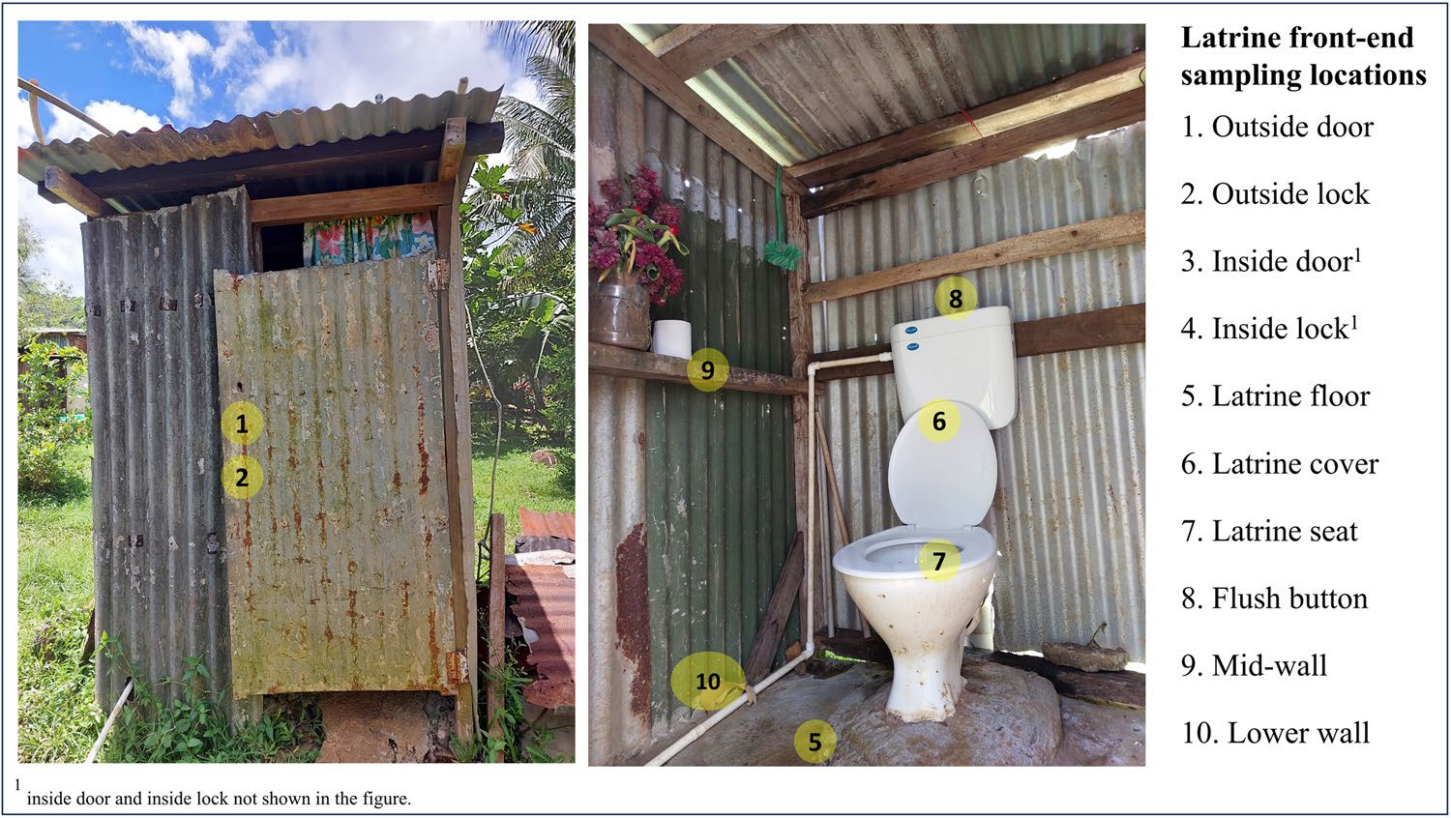
In-depth front-end sampling locations of a cistern-flush latrine at Natadradave, Dawasamu, catchment. The yellow circles with numbers represent the sampling locations

During the in-depth front-end study, swab samples were collected from several surfaces from 12 private household latrines in Natadradave, Dawasamu. Latrines were selected to include different front-end types that are representative of those found within the catchment, including cistern flush, pour-flush and hole-type (pit) latrines. Surface swab samples were collected from nine frequent human contact surfaces, such as outside and inside door handles, outside and inside lock handles, latrine floor, latrine seat, latrine cover, flush button and mid-wall (wall area around the pedestal that is likely to be touched during latrine use (Fig. 2, Table S2).

Furthermore, swab samples were also collected from the lower wall (control), as users are less likely to touch this area. The *E. coli* density on frequent contact surfaces was compared for the 12 private latrines. In total, 97 swab samples were taken from the 12 latrines. Sterile cotton swabs pre-moistened with sterile distilled water were used to collect the samples, as described by Exley et al. (2015). A sterile 25-cm^2^ aluminium foil template (5 cm × 5 cm) was placed over the sampling area, and the swab was held at a slight angle and moved 20 times horizontally and perpendicularly. For small surfaces such as lock handles, the whole area was swabbed. Swabs were cold transported on ice to the field laboratory located nearby (a 10-min drive) and processed within 6 h.

#### Sample analysis

Each swab sample was vortexed in 10 ml of sterile distilled water for 1 min. The swab tip was then squeezed against the tube wall to extract maximum moisture and removed aseptically. The volume of sample solution used for filtration ranged between 1 and 10 ml, depending on the surface contamination. For example, two volumes were plated (1 ml and 10 ml) for samples taken from surfaces with expected high faecal contamination such as latrine floors and seats. The swab liquid was then filtered through a 47-mm filter with a pore size of 0.45-µm filter using the membrane filtration technique. The filter was placed onto the petri dish with m-ColiBlue24® (HACH, USA) and incubated at 37 °C for 24 h (HACH 2023). Blue colonies of *E. coli* were counted, and the density was reported as a colony-forming unit (CFU) per 25 cm^2^ of the surface swabbed. For the inside and outside lock handles, *E. coli* density was reported as CFU per swab. The lower limit of detection was one CFU per plate, equivalent to 1 CFU/ 25 cm^2^. For non-detects or samples below the detection limit, half of the lower limit of detection value was substituted as in similar previous studies (Mraz et al. 2023; Pickering et al. 2018).

#### Quality control

The sample collection, processing and quality control measures were optimised for the limited laboratory and logistic conditions. Daily negative controls were plated by filtering sterile distilled water. Using laboratory-maintained *E. coli* was not feasible for positive control in rural Fiji. Thus, latrine floor samples with expected *E. coli* presence were used, confirming at least 10% positive samples in each batch of daily collected samples (30 samples). A similar method of swabbing latrine surfaces was undertaken for positive control in rural settings (Uprety et al. 2020). The filtration unit was dried and sterilised by burning methanol following WagTech® Potalab + M protocol (Palintest 2023). Samples from visibly clean surfaces were processed before those from dirty surfaces. Working surfaces and hands were frequently sterilised with ethanol to prevent cross-contamination.

### Data analysis

#### Visual assessment of latrine front-end photos

The latrine front-ends were classified primarily by existing flush type (cistern-flush, pour-flush and hole-type) and sitting positions (pedestal or squat types). The presence of moisture and dirt on latrine floors was visually assessed using latrine front-end photos captured during the sanitation observations into three categories: “yes”, “no” and “not differentiated” (Fig. S1). The latrine floors were classified as “yes” if they were visually moist and were classified as “no” if they appeared visually dry, and “not differentiated” if it was not possible to visually differentiate the condition of the floors, either due to inappropriate lighting or photo angles. If the latrine front-end photos were not captured, they were regarded as missing data. Similar classification criteria were applied to assess the presence of dirt on the latrine floors.

#### Statistical analysis

Latrine floor swab samples included in this study were collected during different periods and with no sanitation interventions applied on household levels between these periods. The variability in the mean *E. coli* densities was assessed across the baseline and endline sampling rounds. The Shapiro–Wilk test was used to assess the normality of *E. coli* densities data. Both continuous and log-transformed *E. coli* densities data (*p* < 0.001) were not normally distributed. Therefore, non-parametric tests were used to test the statistical difference between the *E. coli* densities on the latrine surfaces with different latrine characteristics. Specifically, the Mann–Whitney *U* test was used to compare two independent variables, while the Kruskal–Wallis test was used for multiple independent variables. Results were considered statistically significant when *p* ≤ 0.05. All the data analyses were performed using R Studio version 2022.12.0 (RStudio Team 2022). The continuous *E. coli* densities data were purposefully chosen for data visualisation to facilitate easier access and interpretation by a broader non-scientific audience.

## Results

### General characteristics of latrines

Table 1 summarises the latrine front-end characteristics using the survey data from a larger sample size of 311 households from the baseline survey, as similar trends were observed in the endline survey (Table S3). Of 311 households, 216 (69%) had latrines located outside their dwelling, while 95 (31%) had indoor latrines (Table 1). Among the catchments, Dawasamu had the highest percentage of households with latrines located outside (89%), whereas Waibula with the highest for indoor latrines (46%). Regarding latrine ownership, 265 (85%) households had private latrines, while 46 (15%) had shared latrines. Waibula had the highest percentage of private latrines (94%), while Dawasamu had the highest shared latrines (30%).

**Table 1:** Characteristics of latrine front-ends within the five catchments in the baseline survey (311 households)

Catchments	Bureta (55 households)	Dama (65 households)	Dawasamu (56 households)	Upper Navua (65 households)	Waibula (70 households)	Total (311 households)^1^
Latrine location						
Inside	23 (42%)	19 (29%)	6 (11%)	15 (23%)	32 (46%)	95 (31%)
Outside	32 (58%)	46 (71%)	50 (89%)	50 (77%)	38 (54%)	216 (69%)
Latrine ownership						
Private	43 (78%)	58 (89%)	39 (70%)	59 (91%)	66 (94%)	265 (85%)
Shared	12 (22%)	7 (11%)	17 (30%)	6 (9%)	4 (6%)	46 (15%)
Front-end flush type						
Cistern Flush	53 (96%)	38 (58%)	43 (77%)	64 (98%)	61 (87%)	260 (83%)
Pour flush	0 (0%)	23 (35%)	10 (18%)	0 (0%)	6 (9%)	39 (13%)
Hole type (without water seal)	1 (2%)	4 (6%)	3 (5%)	1 (2%)	3 (4%)	12 (4%)
Latrine floor type						
Washable floor	52 (95%)	61 (94%)	50 (89%)	63 (97%)	61 (87%)	287 (92%)
Non-washable floor	3 (5%)	4 (6%)	6 (11%)	2 (3%)	9 (13%)	24 (8%)
Front-end broken						
Yes	22 (40%)	26 (40%)	30 (54%)	23 (35%)	20 (29%)	121 (39%)
No	33 (60%)	39 (60%)	26 (46%)	42 (65%)	50 (71%)	190 (60%)
Anal cleansing material type reported by households^2^						
Toilet paper	29 (53%)	16 (25%)	38 (68%)	28 (43%)	57 (81%)	168 (54%)
Newspaper	0 (0%)	1 (2%)	7 (13%)	0 (0%)	1 (1%)	9 (3%)
Toilet paper and newspaper	24 (44%)	24 (37%)	11 (20%)	37 (57%)	9 (13%)	105 (34%)
Toilet paper and water	0 (0%)	23 (35%)	0 (0%)	0 (0%)	3 (4%)	26 (8%)
Observation of handwashing facilities inside latrines^3^						
Yes	5 (9%)	9 (14%)	1 (2%)	5 (8%)	4 (6%)	24 (8%)
No	49 (89%)	56 (86%)	55 (98%)	60 (92%)	66 (94%)	286 (91%)

^1^Sum of households does not add up to the total number of households as some households had shared latrines

^2^Two missing data

^3^One missing data

**Fig. 3:**
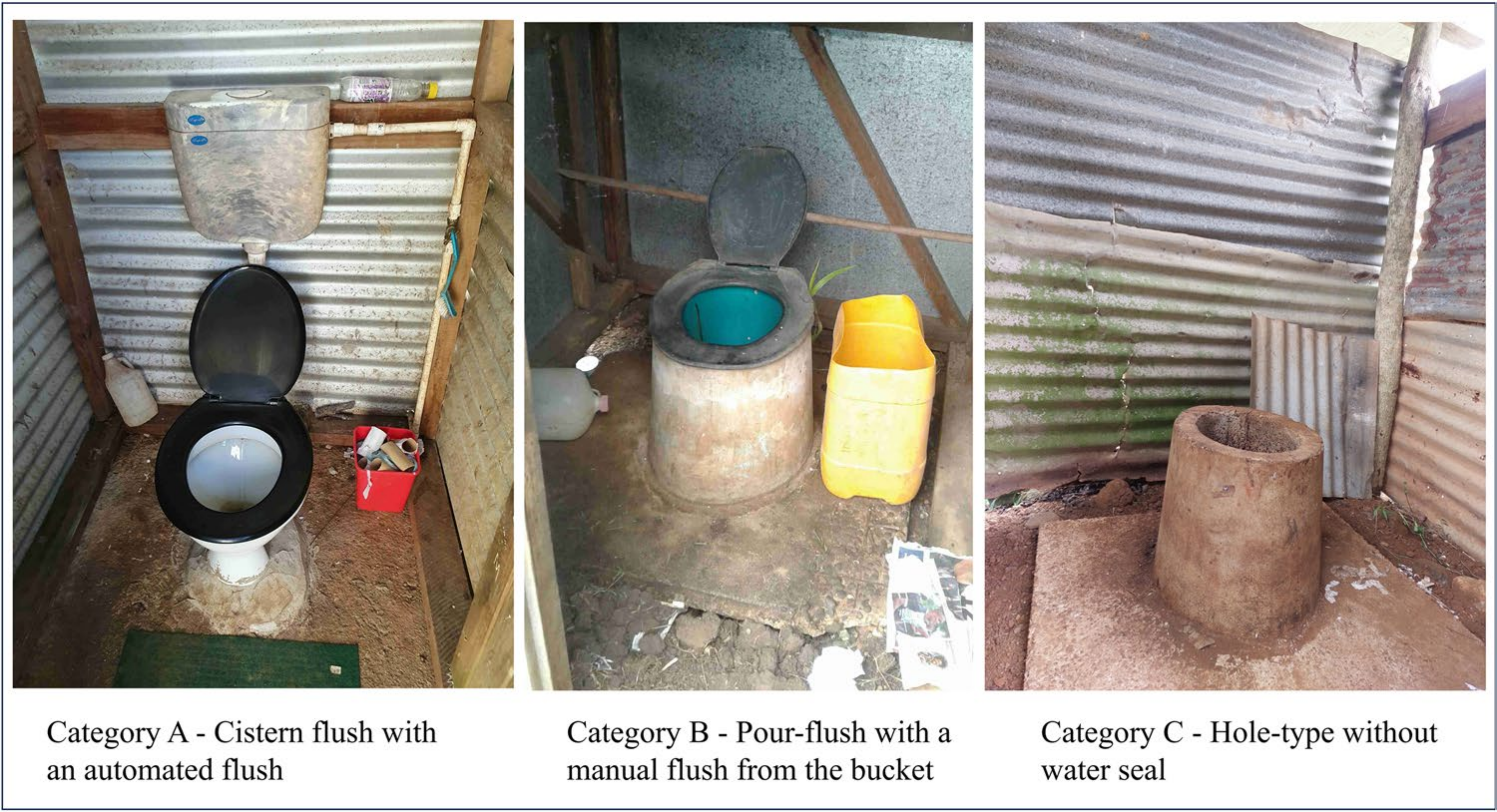
Latrine front-end types based on flush mechanisms observed in rural Fiji

The most common front-end flush type observed was Category A—cistern flush latrines in 260 (83%) households, followed by Category B—pour-flush latrines in 39 (13%) and Category C—hole-type latrines in 12 (4%) households (Fig. 3). Upper Navua had the highest percentage of cistern-flush latrines (98%), while Dama had the highest percentage of pour-flush (35%) and hole-type latrines (5%). Almost all latrines were pedestal-type, except for one squat-type latrine in Upper Navua. For latrine floor type, 287 (92%) households had latrines with washable floors such as coarse concrete and tiles, while 24 (8%) had non-washable latrine floors made of wood and dirt.

Considering latrine front-end maintenance, 121 (39%) households had a broken front-end (broken flush, pipe, seat, or floor), with the highest percentage in Dawasamu (54%) and the lowest in Waibula (29%). Overall, broken front-ends decreased by 12% across all catchments between the baseline and endline survey, with a notable 27% reduction in Dama. The visual assessment of latrine front-end photos showed 130 (42%) with moist latrine floors and 91 (29%) with dry floors, and 89 (21%) could not be differentiated (Table S3). Similarly, 118 (47%) had visible dirt on the latrine floor, 91 (29%) did not have visible dirt and 89 (21%) could not be differentiated.

For anal cleansing materials, 168 (54%) reported using toilet paper only, nine (3%) newspaper only, 105 (34%) both toilet paper and newspaper and 26 (8%) both toilet paper and water. The visual assessment of front-end photos showed 43 (14%) households using latrines to store agricultural tools such as insecticide sprays and jerry cans. Faeces were observed in or around the latrines of 13 (4%) households, of which 12 (92%) had human faeces and 1 (8%) had animal faeces. In terms of child faeces management, 173 (56%) households reported disposing of faeces in the latrines, 38 (12%) threw them together with other solid waste and seven (2%) threw them in rivers. Handwashing facilities were observed inside the latrines of only 24 (8%) households, while 286 (91%) lacked them. However, handwashing facilities were observed elsewhere in 266 (86%) households, with 144 (54%) households with a running water tap and a sink and 71 (21%) with a bucket of water. No handwashing facilities were observed in 45 (14%) households. Regarding handwashing after defecation, 302 (97%) reported washing their hands, but only 142 (46%) reported always using soap. Similarly, only 35 (11%) households reported always wearing shoes outdoors, while 62 (20%) never used them. Considering that 69% of the latrines are located outside households, it is likely that at least some members of these households use latrines without wearing shoes. Only 48 (15%) households reported a household member having diarrhoea in the past month.

During the in-depth front-end study including 12 households, latrine cleaning materials were observed in only four (33%) households, with three having only a brush, and one using a commercial liquid cleaner. None of the latrine cleaning materials were observed in the remaining eight (67%) households. For the latrine cleaning frequency, three (25%) households reported cleaning daily, two (17%) cleaned once a week, six (50%) cleaned twice a week, and one (8%) cleaned thrice a week. The latrine cleaning materials used varied: seven (58%) reported using laundry detergent; one (8%) used a commercial liquid cleaner; two (17%) used ash and water; and two (17%) used only water. For child faeces management, only six (50%) had children, and all of them reported to use diaper. Of these households, three reported washing the diapers in the standpipe and using the remaining diaper plastic to ignite the cooking places, while one reported disposing of diapers in the latrine pit, one burying them in the pit, and one disposing in a rubbish bin with other solid waste. For menstrual hygiene management practices, information was obtained from eight (67%) households, as the remaining four (33%) had male respondents. Among those, four (50%) reported using commercially available disposable pads, one (13%) used reusable folded cloth, and three (37%) used both. Similarly, for disposal of menstrual hygiene materials, 4 (50%) households reported throwing them in a rubbish bin with other solid waste, 3 (37%) threw them in the pit of a hole-type latrine, and one (13%) threw away in the bush.

### Comparison of latrine floor *E. coli* density with latrine front-end characteristics and maintenance in baseline and endline studies

**Fig. 4:**
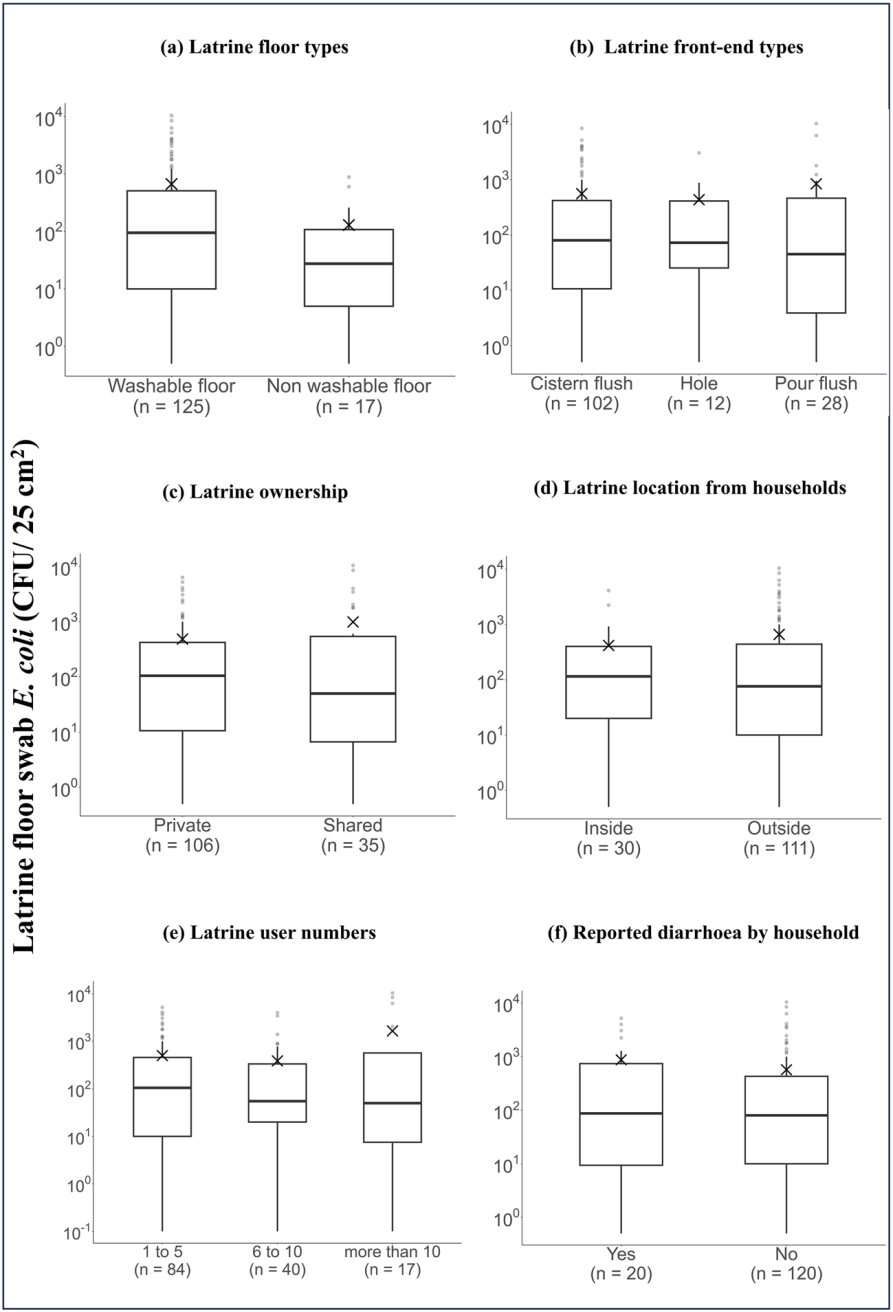
*E. coli* density on latrine floors (CFU/25 cm^2^) with a total of 142 latrine floor swabs from baseline and endline study compared to latrine front-end characteristics. The box plot represents the median, quartiles, outlier (grey dots) and mean ( ×). **a** *E. coli* and type of latrine floors; **b** *E. coli* and latrine front-end flush type; **c** *E. coli* and latrine ownership (one missing data); **d** *E. coli* and latrine location with reference to households (one missing data); **e** *E. coli* and the number of users per latrine (one missing data); **f** *E. coli* and reported diarrhoea by the household in the previous month (two missing data). The lower limit of detection is 1.0 CFU/25 cm^2^. Non-detects were substituted with half of the lower limit of detection values. The *E. coli* densities data presented are raw continuous data

A total of 142 latrine floor swab samples were collected from baseline (96 samples) and endline (46 samples) sampling rounds. The mean *E. coli* density on the latrine floors was found to be comparable with 5.8 × 10^2^ CFU/25 cm^2^ in baseline and 6.6 × 10^2^ CFU/25 cm^2^ in endline (*p* = 0.24) (Fig. S2). Latrines with washable floors were found to have significantly higher *E. coli* density (6.7 × 10^2^ CFU/25 cm^2^) than those with non-washable floors (1.3 × 10^2^ CFU/25 cm^2^) (*p* = 0.05) (Fig. 4a). There were slight variations between the mean *E. coli* densities on the latrine floor by latrine type: pour-flush 8.4 × 10^2^ CFU/25 cm^2^, cistern-flush 5.6 × 10^2^ CFU/25 cm^2^ and hole-type 4.3 × 10^2^ CFU/25 cm^2^ (Fig. 4b). These differences were not statistically significant (*p* = 0.77), likely due to the large range in values. Similarly, there was no statistical difference in the mean *E. coli* densities on the latrine floor for variables such as latrine ownership, latrine location, number of latrine users and reported diarrhoea by households (Fig. 4c to f). There was no statistical difference in the overall mean *E. coli* densities on the latrine floor for the highest education level attained by the households; primary (9.0 × 10^2^ CFU/25 cm^2^), secondary (5.3 × 10^2^ CFU/25 cm^2^) and tertiary level (5.4 × 10^2^ CFU/25 cm^2^) (*p* = 0.07) (Table S4). However, pair-wise comparisons revealed that households with primary education had significantly higher *E. coli* densities on latrine floors than those with secondary (*p* = 0.04) and tertiary education levels (*p* = 0.04). There was no statistical difference in mean *E. coli* densities on the latrine floor with front-end maintenance variables such as visible moisture, visible dirt, broken front-ends and the observation of faeces in or around the latrines (Table S4).

### In-depth front-end study

#### Comparison of *E. coli* density on frequent contact surfaces of latrine front-ends

**Fig. 5:**
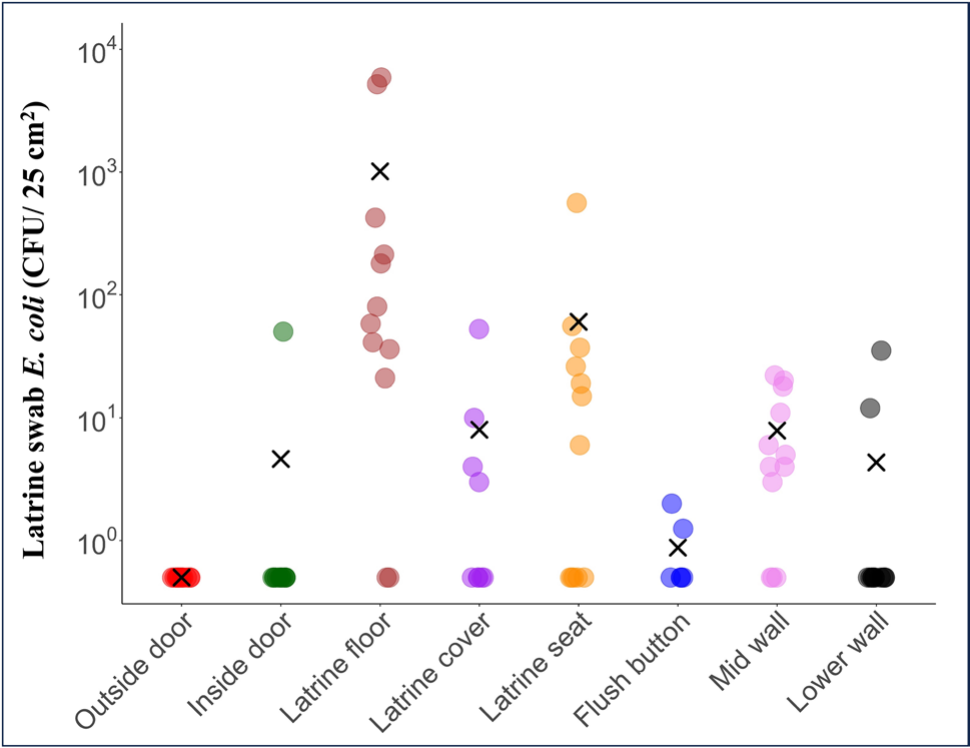
*E. coli* density (CFU/ 25 cm^2^) on various sampling locations of 12 household latrines, including outside door (*n* = 12), inside door (*n* = 12), latrine floor (*n* = 12), latrine cover (*n* = 9), latrine seat (*n* = 12), flush button (*n* = 6), mid-wall (*n* = 12) and lower wall (*n* = 12). The mean is represented by ( ×) for each surface. The lower limit of detection is 1.0 CFU/25 cm^2^. Non-detects were substituted with half of the lower limit of detection values. The *E. coli* density data presented are raw continuous data

A total of 97 swab samples were taken from 10 sampling locations within 12 private household latrines in Natadradave, Dawasamu. All latrines were pedestal types, including six cistern-flush, three pour-flush and three hole-type latrines. Latrine floors had the highest frequency of positive *E. coli* samples, with 83% (10 out of 12 samples) testing positive. Similarly, 75% of mid-wall samples (nine out of 12 samples), 58% of latrine seat samples (seven out of 12 samples), 44% of latrine cover samples (four of nine samples), 20% of outside lock handle samples (four out of five samples) and 8% of inside door samples (one out of 12 samples) were positive for *E. coli* (Table S5). None of the 12 outside door and five inside lock handle samples was positive for *E. coli.* Figure 5 shows *E. coli* densities in all sampling locations, except for outside and inside locks which are provided in Table S5. The latrine floor had significantly high *E. coli* densities (1.1 × 10^3^ CFU/25 cm^2^) compared to the latrine seat (60.0 CFU/25 cm^2^) (*p* = 0.02), latrine cover (7.8 CFU/25 cm^2^) (*p* = 0.007) and mid-wall (7.8 CFU/25 cm^2^) (*p* = 0.004) (Fig. 5).

Within the front-end types, the floors of pour-flush latrines were significantly contaminated with *E. coli* (3.8 × 10^3^ CFU/25 cm^2^) compared to cistern flush (94.0 CFU/25 cm^2^) and hole-type latrines (91.0 CFU/25 cm^2^) (*p* = 0.05) (Table S5). For mid-wall samples, cistern-flush and pour-flush latrines had the same *E. coli* densities (10.0 CFU/25 cm^2^), whereas hole-type latrines had lower densities (1.3 CFU/25 cm^2^), but not statistically significant (*p* = 0.17). There was no significant difference (*p* = 0.46) in *E. coli* densities from the seats of cistern-flush latrines (97.5 CFU/25 cm^2^), hole-type (26.0 CFU/25 cm^2^) and pour-flush latrines (18.5 CFU/25 cm^2^) (Table S5).

#### Comparison of *E. coli* density on frequent contact surfaces with latrine front-end maintenance

Out of the 97 surface samples, only 10 (10%) had visible moisture, and 87 (90%) were dry. Surfaces with visible moisture had significantly higher *E. coli* densities (1.2 × 10^3^ CFU/25 cm^2^) compared to the dry surfaces (14.3 CFU/25 cm^2^) (*p* < 0.001) (Fig. S3 a). Similarly, 15 out of 97 surfaces (15%) had visible dirt. Surfaces with visible dirt had significantly higher *E. coli* densities (8.5 × 10^2^ CFU/25 cm^2^) compared to the clean surfaces (16.5 CFU/25 cm^2^) (*p* < 0.001) (Fig. S3 b). These results highlight that moisture and dirt on latrine surfaces could influence *E. coli* densities on the latrine surfaces.

For surface materials, 54 out of 97 surfaces (56%) were rough (coarse concrete and wood), and 44 (44%) were smooth (plastic, tiles, galvanised iron sheets and rubber). Rough surfaces had significantly higher *E. coli* densities (280.0 CFU/25 cm^2^) compared to smooth surfaces (26.4 CFU/25 cm^2^) (*p* = 0.008) (Fig. S3 c). This underscores that the surface properties of the materials within the latrine front-ends can impact the *E. coli* densities on these surfaces. Similarly, 68 out of 97 surfaces (70%) were washable (including plastic, tiles, rubber, galvanised iron sheets and coarse concrete), and 29 (30%) were non-washable surfaces (wooden and dirt). Washable surfaces exhibited higher *E. coli* densities (223.0 CFU/25 cm^2^) as opposed to non-washable surfaces (6.8 CFU/25 cm^2^), but the difference was not statistically significant (*p* = 0.42) (Fig. S3 d).

## Discussion

### Latrine usage behaviour and associated faecal contamination levels

This study attempts for the first time to investigate the detailed characteristics of latrine front-ends and latrine usage behaviours in rural Fiji. The latrine infrastructure summary for households was private latrines (85%), located outside the main house (69%), predominantly pedestal-type with mostly cistern-flush latrines (83%) and few hole-type latrines (4%). High water availability supports pedestal cistern flush latrines as detailed in six Fijian community focus group findings by Nelson et al. (2022b). Further, cistern flush pedestal latrines are the predominant sanitation preference in the Pacific as people upgrade from pit latrines (Fleming et al. 2019; White et al. 2020). One challenge with cistern flush pedestal latrines is a potential mechanical failure as observed in this study, with 27% of households still reporting issues during the endline surveys. Notably, Sanitation Safety Planning effectively catalysed a 12% reduction in latrine front-end dysfunction across all communities with a notable reduction of 27% in Dama from baseline to endline survey. Considering that this project could not complete contracted latrine infrastructure work, all reductions were due to households’ investment and skills.

Our study found significant five-times higher *E. coli* densities on washable floors compared to non-washable floors. In contrast, Pickering et al. (2012) reported lower (not statistically significant) *E. coli* densities on washable concrete slabs compared to non-washable dirt floors in Tanzania. While Pickering et al. (2012) did not mention the moisture condition of latrine floors, they found a positive correlation between *E. coli* density and moisture content on overall surfaces and soil. Moisture availability is favourable for the survival of microorganisms in environments such as soil and surfaces (Scoullos et al. 2019; Sinclair and Gerba 2011). Thus, the elevated *E. coli* densities on washable floors in Fiji can be attributed to moisture and accumulated dirt, particularly prevalent as 69% of latrines are located outside, increasing the likelihood of dirt and water introduced through user’s feet or footwears, especially in rainy conditions. Also, nearly half of the latrine floors showed visible moisture and dirt during baseline and endline surveys, resulting to higher *E. coli* densities compared to dry and clean floors (Table S4).

The in-depth study revealed significantly higher *E. coli* densities on pour-flush latrine floors compared to cistern-flush and hole-type floors. However, no significant difference was found across different front-end types when comparing 142 latrine floor samples from baseline and endline studies, possibly due to large variations in sample sizes among the front-end types. While comprehensive research with a larger sample size is needed to confirm this association, it can be deduced that pour-flush floors are likely to be moist from manual flushing from buckets, irrespective of pedestal or squat types. This aligns with findings from rural Cambodia reporting elevated *E. coli* on pour-flush floors and squat plates (75.0 CFU/25 cm^2^) (Sinclair and Gerba 2011). Furthermore, water leakages were common in cistern flush latrines in Fiji, with some households using them as pour-flush without repairing the flush system (Fig. S4 a). Moist latrine fabric floor mats beneath the cistern and pour-flush pedestals further contributed to moisture retention (Fig. S4 b).

The elevated *E. coli* levels on washable latrine floors in Fiji could also be from child defecation practices. This is deducted as 56% of the households reported disposing of the child faeces in latrines, and faeces were observed in or around latrines of some households (4%). Although it was not captured in the survey, co-authors confirmed it as a common practice in rural Fiji where children initially defecate on the latrine floor, which is then scooped by mothers using toilet paper and disposed of in latrines. Similar practices have been documented in Indonesia (Agestika et al. 2022) and India (Routray et al. 2015). Thus, inadequate cleaning and disinfection of latrine floors can exacerbate *E. coli* levels. Also, cleaning rough latrine surfaces such as coarse concrete floors commonly used in rural Fiji can be challenging. These surfaces can easily accumulate dirt and moisture in small cavities which is evident by significantly higher *E. coli* densities compared to smooth surfaces in our study (Fig. S3 c). Without consistent use of effective cleaning products, disinfecting these surfaces becomes challenging. Previous studies have also highlighted the difficulty of cleaning rough surfaces, resulting in unhygienic and unpleasant odours in latrine front-ends (Crofts and Fisher 2012; Ishida et al. 2021; Stenström et al. 2011). Thus, ensuring that latrine surfaces are user-friendly, easy to clean and disinfected is crucial (Jaglarz 2020).

The significant variation in *E. coli* densities on the latrine floor between households with primary education and those with secondary or tertiary education in our study aligns with Exley et al. (2015) in Tanzania, where higher household education levels were associated with lower *E. coli* densities on frequent contact surfaces of latrines. While higher household education levels could lead to cleaner latrines due to increased awareness of the disease burden of poor sanitation and hygiene (Exley et al. 2015; Temesgen et al. 2021), other socio-economic variables such as household income and occupation could also influence this outcome. Regarding the frequent hand contact surfaces, mid-wall areas were more frequently contaminated (75% of samples positive) compared to latrine seats and covers, suggesting potential contamination through hand contact with the mid-wall area while fetching anal cleansing papers. It could also be linked to less attention given to mid-wall areas while cleaning latrines. Latrine surfaces such as door handles, lock handles and flush buttons showed low to no *E. coli* detection. Fijian households commonly used dry anal cleaning (toilet paper and newspapers), and the majority lacked handwashing facilities inside latrines, reducing moisture on these surfaces. This aligns with a previous study in the UK (Mendes and Lynch 1976). The *E. coli* contamination on the cistern and pour-flush latrine surfaces could also be from the deposition of flush-generated aerosols (Luo et al. 2023). Although we did not sample the surfaces of containers and agricultural tools stored in latrines (Fig. S4 c), their proximity to the latrine pedestal suggests potential contamination from flush-generated aerosols (Goforth et al. 2024).

### Latrine surfaces contamination and its implication for infection risks in rural Fiji

The absence of the recommended surface hygiene standards for bacterial densities on latrine surfaces creates challenges on what to consider a clean surface (Hambraeus and Malmborg 1980; Leas et al. 2015). *E. coli* densities observed on different latrine surfaces in our study heighten the infection risks to rural Fijians as poorly maintained latrine infrastructures and cleanliness have previously been linked to significant infection risks (Adane et al. 2017; Beyene and Melku 2018; Dumba et al. 2008). There was no significant association between latrine floor *E. coli* densities and households reporting diarrhoea, suggesting that latrine floors might not be the primary route for diarrheal transmission in Fiji. Other factors such as food hygiene, contaminated water and poor hygiene might contribute to faecal-oral diseases in Fiji (Nelson et al. 2022a; Prasad et al. 2018). However, there is a significant risk of the pathogen transfer from latrine floors to other areas of households such as the yard and kitchen (Stenström et al. 2011). Latrine floors, with substantial moisture and dirt, facilitate pathogen survival and growth, including soil-transmitted helminths (STHs) (Dumba et al. 2008; Hassan and Oyebamiji 2018). While our study did not measure STH densities, prior research has reported them on (Schulz and Kroeger 1992) latrine floors (Baker and Ensink 2012; Exley et al. 2015). This risk is further exacerbated considering that only 11% of households in our study reported always wearing shoes outdoors, and inconsistent outdoor shoe usage has been associated with an increased risk of STHs in Fiji (Kim et al. 2020).

Previous studies highlight the efficient transfer of pathogens from contaminated hard and non-porous surfaces to hands, with elevated transfer rates from moist surfaces (Lopez et al. 2013; Rusin et al. 2002). This is relevant in Fiji, where latrine seats and mid-wall surfaces are made of hard and non-porous surfaces such as plastic and corrugated galvanised sheets. Properly cleaned latrine surfaces generally pose lower risks; however, circumstances such as the illness of family members or surfaces with visible faeces increase the infection risk significantly (Bloomfield et al. 2012). Surfaces such as latrine seats pose transmission risks not only through hand contact but also from the exposed skin during latrine usage (Jeon et al. 2013). Therefore, regular cleaning and disinfection of latrine surfaces, even with low contamination levels, are vital for protecting vulnerable household members, such as younger children, pregnant women, the elderly and individuals with compromised immune systems (Ojima et al. 2002; Potgieter et al. 2020). Given that 92% of the households in our study lack handwashing facilities within the latrines and 14% have no handwashing facilities available, this further increases the microbial risk from latrine surfaces via unwashed hands. Only 46% of households reported always using soap during handwashing after defecation, which reveals a major gap in hygiene practices. Considering the evidence that frequent handwashing after defecation lowers the risk of typhoid fever in Fiji (Prasad et al. 2018), more interventions are needed to promote proper and sustainable handwashing practices.

This study addresses a significant data gap in sanitation literature for the Pacific regions by quantifying the field-based microbial densities on latrine surfaces in rural Fiji. The current literature lacks quantitative risk assessment approaches for potential health risks from latrines (Gwenzi et al. 2023). Previous studies have consistently emphasised the paucity of published data on pathogen density on latrine surfaces, limiting the application of quantitative microbial risk assessments (Abney 2022; Adhikari et al. 2023; Bloomfield et al. 2012). While we only measured *E. coli*, other studies have reported various pathogens on household latrine surfaces, including bacteria; *Clostridium difficile* (Kim et al. 1981), *Staphylococcus aureus* (Medrano‐Félix et al. 2011), *Salmonella* spp. (Barker and Bloomfield 2000); and viruses such as influenza A (Boone and Gerba 2007) and helminth eggs (Schulz and Kroeger 1992). Some pathogens have extremely low infective doses such as pathogenic strains of *E. coli* and *Shigella* spp. (less than 10 CFU) (Kothary and Babu 2001; Schmid-Hempel and Frank 2007) and norovirus (10 to 100 particles) (Yezli and Otter 2011), highlighting the potential transmission risk via contaminated latrine surfaces (Barker and Bloomfield 2000; Hossain et al. 2021). Therefore, our findings are important in guiding the subsequent quantitative risk assessment steps in determining the probability of microbial infection risk from latrine surfaces.

### Study limitations

In the baseline survey, the random sampling approach for selecting 311 households across 29 communities might not have represented all latrine front-end types in proportion to their actual distribution. Moisture on the latrine floor was visually assessed through front-end photos in the baseline and endline survey; thus, some could not be differentiated due to improper photo angle and missing photos. However, these factors were considered during the in-depth front-end study. Latrine floor samples in the baseline and endline study were collected over 4 months, covering dry and wet seasons. Despite this difference, the mean *E. coli* densities across both sampling rounds were consistent, allowing for comparative analysis, likely due to minimal seasonal variations in temperature and rain days in our study locations in Fiji (Kumar et al. 2014; Sharma et al. 2021). Future studies should control for environmental variables such as temperature, moisture and rainfall to understand their impact on *E. coli* densities on latrine surfaces, particularly in regions with pronounced seasonal variations. In addition, investigations of *E. coli* recovery efficiency from different material surfaces, which was beyond the scope and feasibility of this study, may be warranted to inform future comparisons of latrine front-end types. Regardless of those results, the current observed high *E. coli* densities on latrine surfaces still pose potential health risks to households. While household education level was analysed, this study did not analyse or control for other socio-economic variables, such as household income level and occupation, which might have influenced the results. Household income data were not collected across all the studied households, and the majority of the rural Fijian in studied households engaged in agriculture as their main occupation.

The in-depth front-end study was limited to one community with a small sample size, so it might not represent all other rural Fijian communities. Our study included only one sample from each latrine surface and was limited to conducting serial dilutions; thus, sample results included non-detects. This can be improved by collecting duplicates or triplicates, if not limited by resources and logistics. We only measured *E. coli* as a faecal indicator organism using the traditional culture method, which can have both human and animal sources. Future exploration using molecular microbiology is warranted to identify the sources of *E. coli* on latrine surfaces. Potential bias exists as households might have cleaned their latrines in anticipation of sampling visits, influencing the observed *E. coli* densities. For the statistical analysis, while we used non-parametric tests, the sample size varied largely across the categories that need to be considered when interpreting the results.

## Conclusion and recommendations

This is the first study to assess faecal contamination levels on latrine front-end surfaces in rural Fiji. Our findings highlight that the surfaces of latrines considered more protective on the sanitation ladder (cistern-flush or pour-flush latrines) and with washable floors had higher faecal contamination compared to surfaces of latrines at a lower position of the sanitation ladder (hole-type latrines) and non-washable floors. It is imperative to stress consistent cleaning, disinfection and maintaining dry latrine floors, even when washable surfaces are used. Despite the commendable coverage of flush latrines in rural Fiji, our study emphasises that latrine cleanliness and hygiene are as critical as latrine infrastructure for effectively disrupting faecal pathogens and reducing faecal-oral diseases such as typhoid in Fiji. Therefore, the availability of appropriate and affordable cleaning agents should be ensured in communities. Safe child faeces management practices such as using portable potties to dispose of faeces in latrines, need to be promoted. Designated footwear for latrine usage can minimise pathogen transmission from latrine floors. This study emphasises the urgency of educating communities on handwashing with soap and the infrastructure maintenance of the latrine front-end. These recommendations can reduce the overall health risk associated with sanitation in Fiji and hold relevance to other Pacific regions and countries with similar challenges in sanitation globally.

Future research could quantify microbial densities of specific pathogens (such as viral, bacterial and helminths) on latrine surfaces, considering material properties such as surface roughness and porosity. Such details can inform the design of user-friendly front-end components that are easy to clean and disinfect. Further research can keep the latrine front-end type constant with varying moisture and dirt on latrine surfaces to determine the direct relationship between latrine front-end types and *E. coli* densities. Studies with larger sample sizes covering diverse geographical regions can better control for environmental and household socio-economic variables. Additional research can quantify the contribution of contaminated latrine surfaces to faecal-oral disease transmission compared to other pathways, such as flies, contaminated drinking water and other environmental factors. Extending this research to different countries with distinct front-end types (for example, squat-type latrines) and anal cleansing methods (for example, anal washing) can inform targeted interventions to effectively reduce the microbial risks from latrines.

## Supplementary Information

Below is the link to the electronic supplementary material.

Supplementary file1 (DOC 1.02 MB)

## Data Availability

The authors declare that the data supporting the findings of this study are available within the paper's supplementary materials.
