# Supplementary material for: An assessment of latrine front-end characteristics and associated surface E. coli indicated faecal contamination in rural Fiji
Source: Environ Sci Pollut Res Int. 2024 Aug 21;31(40):52948–62. doi: 10.1007/s11356-024-34668-x (PMC11379791; doi:10.1007/s11356-024-34668-x)
Supplement: Supplementary file 1 — Supplementary file1 (DOC 1.02 MB) [file 11356_2024_34668_MOESM1_ESM.doc]

Research Article

**An assessment of latrine front-end characteristics and associated surface *E. coli* indicated faecal contamination in rural Fiji**

Sabita Adhikari ^1^*, Shylett Anthony ^2^, Ponipate Baleinamau ^2^ Jeremaia Coriakula ^2^, Thompson Daurewa ^2^, Rachel Devi ^2^, Sikeli Gavidi ^2^, Pierre Horwitz ^3^, Erin C. Hunter ^4^, Aaron Jenkins ^3,5^, Stacy Jupiter ^6^, Maria Lalamacuata ^2^, Kinikoto Mailautoka ^2^, Sangeeta Mangubhai ^7,8^, Kelera Naivalu ^2^, Timoci Naivalulevu ^2^, Vilisi Naivalulevu ^2^, Nabeela Nasim^1^, Sikeli Naucunivanua ^7^, Joel Negin ^5^, Paul van Nimwegen ^7^, Anaseini Ratu ^5^, Mereia Ravoka ^7^, Andrew Tukana ^7^, Jack van de Vossenberg ^9^ , Donald Wilson ^2^, and Jacqueline Thomas ^1^

1. School of Civil Engineering, The University of Sydney, Darlington, NSW 2006, Australia (sabita.adhikari@sydney.edu.au, nabeela.nasim@sydney.edu.au, jacqueline.thomas@sydney.edu.au)
2. Fiji Institute of Pacific Health Research, College of Medicine, Nursing & Health Sciences, Fiji National University, Hoodless House, Suva, Fiji (shylettanthony@gmail.com, ponipate.baleinamau@fnu.ac.fj, jeremaiacoriakula@gmail.com [daurewathompson@gmail.com](mailto:daurewathompson@gmail.com), gavidisikeli1@gmail.com, lalamacuatasia@gmail.com, mailautoka.kinikoto@gmail.com, kelera.tuisova@gmail.com, timoci.naivalulevu@fnu.ac.fj, vratukalou@gmail.com, donald.wilson@fnu.ac.fj)
3. Centre for People, Place, and Planet, Edith Cowan University, Joondalup, WA, Australia (p.horwitz@ecu.edu.au, aaron.jenkins@ecu.edu.au)
4. Department of Public Health Sciences, College of Behavioural, Social and Health Sciences, Clemson University, United States (hunter9@clemson.edu)
5. School of Public Health, Faculty of Medicine and Health, The University of Sydney, Camperdown, NSW 2006, Australia (erin.hunter@sydney.edu.au, aaron.jenkins@sydney.edu.au, joel.negin@sydney.edu.au, anaseini.ratu@sydney.edu.au)
6. Wildlife Conservation Society, Melanesia Program, Suva, Fiji (sjupiter@wcs.org)
7. Wildlife Conservation Society, Fiji Program, Suva, Fiji (smangubhai@gmail.com, sikelinaucunivanua@adra.org.fj, pvannimwegen@wcs.org, mravoka@wcs.org, andrewtukana15@gmail.com)
8. Talanoa Consulting, 28 Disraeli Road, Suva, Fiji (smangubhai@gmail.com)
9. Water Supply, Sanitation and Environmental Engineering Department, IHE Delft Institute of Water Education, Delft, The Netherlands (j.vandevossenberg@un-ihe.org)

***** Correspondence: Sabita Adhikari; Email: sabita.adhikari@sydney.edu.au; Tel.: +61 293512136

# Supplementary materials

**Table S1**: Sanitation and household survey questionaries and observation checklists for in-depth front-end study

**Table S2**: Details of the sampling locations and the area swabbed in the latrine front-end for in-depth front-end study.

**Table S3**: Latrine front-end characteristics, household sanitation and hygiene behaviours within the five catchments including the baseline (311 households) and endline survey (259 households).

**Table S4:** *E. coli* density (CFU/25 cm^2^) on latrine floor with latrine front-end characteristics in baseline and endline sampling with a total of 142 latrine swab samples.

**Table S5:** *E. coli* density (CFU/25 cm^2^) on various sampling locations in 12 latrines of in-depth front-end study with types of latrine front-end flush systems (cistern flush, pour-flush and hole).

**Fig. S1** Categories for visual assessment of moisture on the latrine floor in rural Fiji

**Fig. S2** *E. coli* density (CFU/25 cm^2^) on the latrine floor from baseline (n = 96) and endline (n = 46) study

**Fig. S3** *E. coli* density (CFU/25 cm^2^) on overall sampling surfaces in 12 latrines of in-depth front-end study with latrine front-end maintenance

**Fig. S4** Latrine usage behaviour observed in rural Fiji. a) Cistern flush latrine used as pour-flush after the cistern is broken; b) Cistern flush latrine with the washable floor (concrete) moist toilet mat; c) Latrine front-end used as storage for agricultural tools and jerry cans

**Table S1**: Sanitation and household survey questionaries and observation checklists for in-depth front-end study

| Household sanitation survey questionaries *√ Tick in the box provided*   1. **Name of village: Date:** 2. **Household code:** 3. **Community type**:  Village (registered)  Fijian settlement Indo- Fijian settlement Mixed Others 4. **Household location:** GPS (Lat): GPS (Long)**:** 5. **Weather (today):**  Sunny  Rainy  Cloudy  Part A – Household survey  1. **Interviewee name:** 2. **Interviewee sex:**  Male Female  Other 3. **What is the highest education level in the household?**  No formal education  Primary (elementary) Secondary (high school) Tertiary (university or technical school) 4. **What is the main occupation of the household? (specify):** 5. **Total family members (number): Male: Female: Others:** 6. **How many children and their ages?**  **Number:** **Ages:** 7. **What is the kind of household ownership?** Owned  Rented  Rent-free  Squatter  Other (specify) 8. **What kind of animals do you have at home and how many?** Dog ( )   Cat **(**  )  Goat ( )  Pig ( )  Horse ( ) Cow( )  Chicken ( )  Duck ( ) None  Others (specify)  *(Mention number in brackets)*   1. **Has any family member suffered from any of any diseases in the past two years?**  Yes  No 2. **If yes, which diseases?** Diarrhea Dysentery Typhoid Helminths Leptospirosis Dengue Others(specify)   **10a. Detail of the family members who suffered**.  Relation: Age: Gender: Name of the disease: Time of infection:    **10b: If more, detail of the family members who suffered**  Relation: Age: Gender: Name of the disease: Time of infection  **10c: If more, detail of the family members who suffered**  Relation: Age: Gender: Name of the disease: Time of infection Part B – Sanitation Survey  1. **When was the toilet built?** ………Years  Don’t know 2. **How many households use this toilet?** Number: 3. **How many people use this toilet?** Number: 4. **How often do you use a latrine?** Always Usually Sometimes Never 5. **What do you use to clean after defaecation?**  Only toilet paper  Only water  Both water and toilet paper  Newspaper  Leaves  Others (Specify) 6. **How do you manage the anal cleansing material?**  Flush in toilet  store in bin inside toilet  store in bin outside toilet  Others (specify): 7. **Where do children defecate?**  Use Potty Use diapers Household yard Others (specify) 8. **How do you dispose of children’s faeces?**  In toilet  with solid waste (rubbish) throw in the bush leave it (do nothing)  others (specify) 9. **How often do you normally wash your hands after using toilets?**  Always  Only after defecation  Usually  Sometimes  Rarely  Never 10. **Do you normally wash your hand in these conditions?**  Before cooking  before eating  before feeding children  after urinating after defecating  after working in the garden after handling children’s faeces  After handling animals   (Tick multiple as applicable)   1. **Is soap used to wash your hands?**  Always Usually Sometimes  Rarely Never 2. **What other material do you use to wash your hands?**  Ash  Only water Other (specify) 3. **Do you use the toilet without slippers/shoes?**  Always  Usually Sometimes  Rarely Never 4. **Do you use separate slippers/shoes for the toilet?**  Yes No 5. **How frequently do you clean your toilet?**  Daily  Once in a week Twice a week  Once in two weeks  Other (specify) 6. **What do you use to clean the toilet?**  Toilet cleaners  Disinfectant  Lime   Ash  Only water Others (specify)   1. **Have you ever repaired your toilet?**  Yes  No 2. **If yes, when was the repair done?** **Time:** 3. **If yes, what kind of repair?**  Flush handle  Cistern  Floor Toilet seat Wall Floor Door  Tap Other(specify)   ***Menstrual hygiene management.***   1. **What is the common material used by females during menstrual hygiene management?**  Commercial/disposable pads  tampons  reusable cotton pads   folded cloth  others (specify)   1. **Where do females in the house dispose the menstrual hygiene management materials?**  Flush in the toilet together in the rubbish bin  dispose of in a separate bin  Throw it away in river  Throw it away in bush  Others (specify) 2. **If reusable menstrual hygiene materials (cotton pads or cloths) are used, where are they washed?** In toilets  in shower  in handwashing station  in the river   others (specify) Sanitation Observation ChecklistsPart C (Toilet front-end features)  1. **Latrine code: Time:** 2. **Location of Toilet**: **GPS (Lat): GPS (Long):** 3. **Location of toilet reference to the household?** Outside  Inside 4. **If outside, what is the distance between the toilet and the house? ………… meters** 5. **What is the toilet backend (connection)?** Municipal sewerage Septic tank Pit Environment Can’t observe 6. **Photo taken? (Toilet door from outside)** 7. **Is the entry to the toilet through the door**?  Yes  No (open entry) 8. **If yes, what is the door made up of?**  CGI sheet Wooden Cloth curtain  Plastic  Others (specify) 9. **Are the door handles present on both sides of the door?** Both sides  Only outside  Only inside  Not present 10. **What is the door handle made of?**  Wooden  Steel  Iron  Others (specify) 11. **Are the door locks present on both sides of the door?**  Both sides  Only outside Only inside  Not present 12. **What is the door lock (out) made of?** Wooden  Iron Steel Others(specify): 13. **What is the door lock (inside) made of?** Wooden  Iron Steel  Others(specify): 14. **Photo taken? (Toilet door from the inside)** 15. **What is the flush mechanism?**  Cistern flush  Pour flush  Dry toilet (hole) Others 16. **What is the type of toilet seat (based on sitting position)?**  Pedestal  Squat Others (Specify) 17. **If pedestal, what is the type of pedestal base?**  Porcelain Plastic Concrete  Wooden Seat covered with plastic  Others (specify) 18. **If pedestal, what is the type of toilet seat?**  Porcelain Plastic Concrete   Wooden seat Seat covered with plastic  Others (specify)   1. **If squat, what is the type of toilet pan?**  Porcelain Plastic  just hole (in ground)  Others (specify) 2. **Photo taken? (Toilet seat)** 3. **What kind of anal cleansing material are present?**  Toilet paper  Water Newspaper Leaves None Others(specify) 4. **Is there water stored in a container inside the toilet?**  Yes  No 5. **Is there a bin for the disposal of sanitary products?** Yes  No 6. **Is there a separate slipper used for the toilet?** Yes No 7. **What is the floor around the footrest area made of (just below the toilet seat)?** Tiles Concrete Wood Dirt others (specify) 8. **What is the floor made of in other areas (besides footrest?** Tiles  Concrete Wood Dirt  others (specify) 9. **Is the floor around the footrest area covered?**  Yes No 10. **If yes, what is the floor covered with?**  Plastic sheet Toilet mat cloth  others (specify) 11. **Photo taken? (Toilet floor)** 12. **What is the lower wall (just 10 cm above the floor) made of**? Concrete  Tile  Wooden  CGI sheets Brick wall Others 13. **What is the upper wall (above 10cm above the floor) made of?**  Concrete  Tile Wooden CGI sheets Brick wall Others(specify) 14. **What is the toilet roof made of?**  CGI sheet  Concrete  Plastic sheet Wood/bamboo Leaves  No roof Others (specify) 15. **Does sunlight enter inside the toilet?**  Yes  No 16. **If yes, which part?**  Floor Wall Other (specify) 17. **Photo taken? (Toilet wall)** 18. **Is there a running water (piped) tap inside the latrine?** Yes (functional) Yes (non-functional) No 19. **Is there a handwashing facility present inside (within the same door) the toilet?**  Yes No 20. **Is there a handwashing facility present within the vicinity the toilet outside?**  Yes No 21. **If yes, what is the distance between the handwashing facility and the latrine?** **……meters** 22. **Are handwashing facilities present elsewhere in the household?**  Yes No 23. **If yes, where?** **Specify:** 24. **What kind of handwashing facilities are present?**  Tap with running water and sink  Bucket with tap  Bucket of water with ladle  Others (specify) 25. **Is hand cleaning material present in the handwashing station?**  Yes No 26. **If yes, what type of material is present?** Liquid soap  Bar of soap  Ash Others 27. **Picture taken?**  Part D (Toilet maintenance)  1. **Is the toilet broken?**  Yes  No 2. **What is broken?**  Seat  Flush  Floor  Pipes Wall Roof other (specify) 3. **Is faeces observed inside the toilet?**  Yes  No 4. **If yes, where?**  In toilet bowl Floor Wall Toilet seat Others(specify) 5. **Is dirt observed in the toilet?** Yes  No 6. **Is the toilet floor moist?**  Yes  No 7. **If yes, where is it moist?**  All floor Around the footrest Towards toilet corners Other(specify) 8. **Any water pooling occurring on the floor?**  Yes  No 9. **Is the lower wall (10cm above the floor) moist?** Yes No 10. **Is there toilet cleansing material present in the toilet?** Yes  No 11. **If yes, what kind of cleaning material is present?**  Brush  Mob Toilet cleaner  None Others 12. **Is there a smell in the toilet front-end?** Yes  No 13. **Is faeces observed in the vicinity of the toilet?**  Yes No 14. **If yes, what type?** Adult Child  Animal 15. **Is the backend receptacle broken?**  Yes  No  Can’t observed 16. **What is broken?** Container  Connection  Effluent pipe  Others (specify) 17. **Is the toilet leaking or overflowing?**  Leaking overflowing No  Can’t observed 18. **Are crops grown in the vicinity of the of toilet?**  Yes No  Can’t observe 19. **If yes, what types?**  Taro  Cassava Leafy greens  Fruit  Other(specify) 20. **If yes, where are the crops relative to the toilet?**  Uphill  Downhill  Same level 21. **Are crops grown in the vicinity of the effluent discharge zone?**  Yes  No Can’t observe 22. **If yes, what types?** Taro  Cassava  Leafy greens Fruit Other(specify)   **If yes, where are the crops relative to the toilet?**  Uphill  Downhill Same level |
| --- |

**Table S2**: Details of the sampling locations and the area swabbed in the latrine front-end for in-depth front-end study.

| **SN** | **Sampling points** | **Sampling area** | **Details of sampling location** |
| --- | --- | --- | --- |
| 1 | Outside door | 5 cm × 5 cm | Door handle on the outside of latrine door. If no door handle was present outside, a potential area on the outside of the door likely to be touched during latrine usage was swabbed |
| 2 | Inside door | 5 cm × 5 cm | Door handle on inside of latrine door. If no door handle was present inside, a potential area on the inside of the door likely to be touched while using the latrine was swabbed. |
| 3 | Outside lock | whole surface | Lock handle on the outside of the latrine door. |
| 4 | Inside lock | whole surface | Lock handle on the inside of latrine door. |
| 5 | Latrine floor | 5 cm × 5 cm | Floor area around the pedestal where the user would place the feet during latrine usage. |
| 6 | Latrine cover | 5 cm × 5 cm | The potential area around the tip of the cover where the user would touch. |
| 7 | Latrine seat | 5 cm × 5 cm | An area where the user would be seated during latrine usage. |
| 8 | Flush button | 5 cm × 5 cm | The flush buttons of cistern flush latrines. |
| 9 | Mid-wall | 5 cm × 5 cm | The potential wall area around the pedestal that is likely to be touched during latrine usage. Usually, the side of the wall where anal cleaning materials (toilet paper or newspaper) were stored. |
| 10 | Lower wall | 5 cm × 5 cm | Latrine wall 10 cm above the latrine floor (control). |


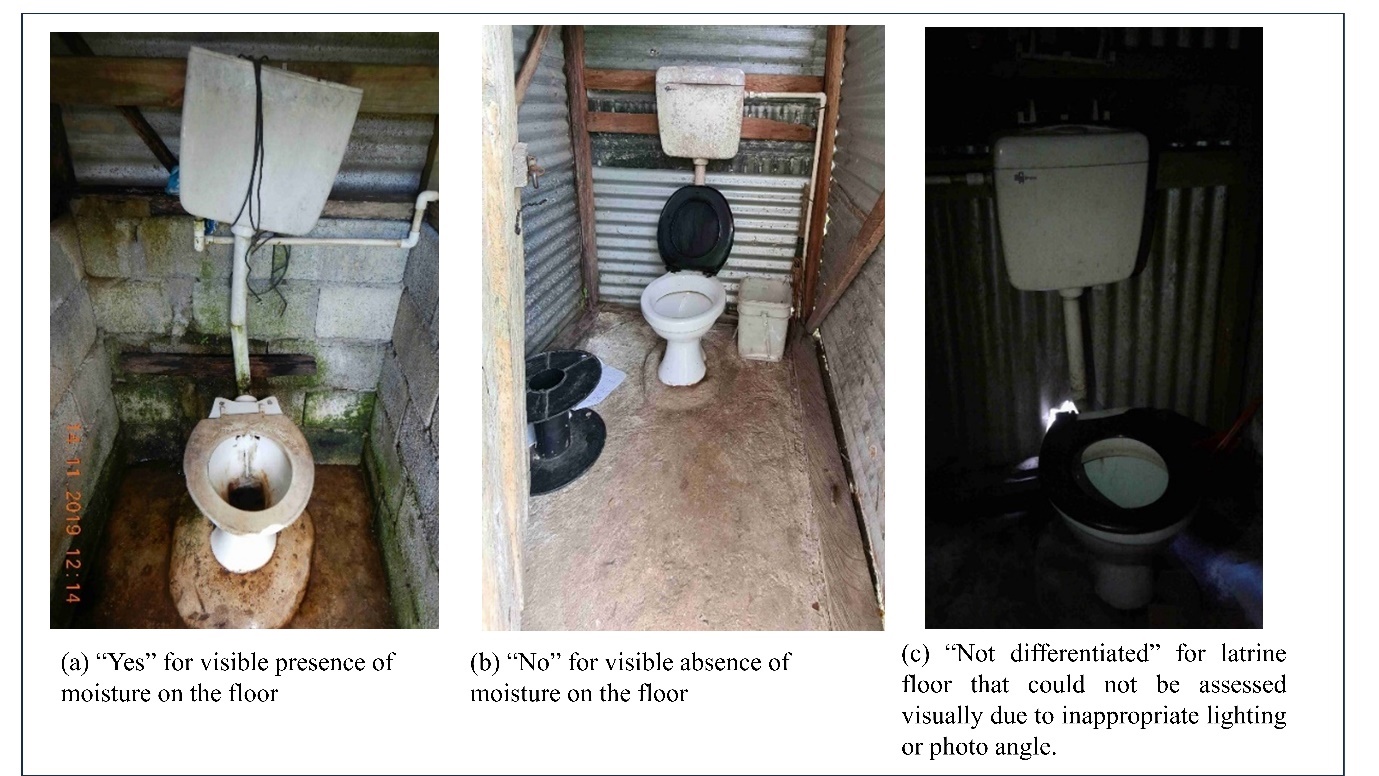


**Fig. S1** Categories for visual assessment of moisture on the latrine floor in rural Fiji

**Table S3**: Latrine front-end characteristics, household sanitation and hygiene behaviours within the five catchments including the baseline (311 households) and endline survey (259 households).

| **Catchments** | **Bureta** |  |  | **Dama** |  |  | **Dawasamu** |  |  | **Upper Navua** |  |  | **Waibula** |  |  | **Total** |  |  |
| --- | --- | --- | --- | --- | --- | --- | --- | --- | --- | --- | --- | --- | --- | --- | --- | --- | --- | --- |
| **Sampling rounds** | Baseline | Endline | % change | Baseline | Endline | % change | Baseline | Endline | % change | Baseline | Endline | % change | Baseline | Endline | % change | Baseline | Endline | % change |
| **No of households** | 55 HHs | 41 HHs |  | 65 HHs | 53 HHs |  | 56 HHs | 52 HHs |  | 65 HHs | 53 HHs |  | 70 HHs | 60 HHs |  | **311 HHs** | **259 HHs** |  |
| **Latrine locations** |  |  |  |  |  |  |  |  |  |  |  |  |  |  |  |  |  |  |
| Inside | 23  (42%) | 18 (44%) | 2% | 19 (29%) | 19 (36%) | 7% | 6  (11%) | 9  (17%) | 6% | 15 (23%) | 17 (32%) | 9% | 32 (46%) | 31 (52%) | 6% | **95 (31%)** | **94 (36%)** | 5% |
| Outside | 32  (58%) | 23 (56%) | 2% | 46 (71%) | 33 (62%) | 9% | 50  (89%) | 42  (81%) | 8% | 50 (77%) | 36 (68%) | 9% | 38 (54%) | 29 (48%) | 6% | **216 (69%)** | **163 (63%)** | 6% |
| **Latrine ownership** |  |  |  |  |  |  |  |  |  |  |  |  |  |  |  |  |  |  |
| Private | 43  (78%) | 33 (80%) | 2% | 58 (89%) | 51 (96%) | 7% | 39  (70%) | 33  (63%) | 7% | 59 (91%) | 49 (92%) | 1% | 66 (94%) | 51 (85%) | 9% | **265 (85%)** | **217 (84%)** | 1% |
| Shared | 12  (22%) | 8 (20%) | 2% | 7  (11%) | 2  (4%) | 7% | 17  (30%) | 18  (35%) | 5% | 6  (9%) | 4  (8%) | 1% | 4  (6%) | 8 (13%) | 7% | **46 (15%)** | **40 (15%)** | 0% |
| **Latrine user number ^1^** |  |  |  |  |  |  |  |  |  |  |  |  |  |  |  |  |  |  |
| 1 to 5 | 37  (67%) | 26  (63%) | 4% | 37 (57%) | 30  (57%) | 0% | 30  (54%) | 21  (40%) | 14% | 47 (72%) | 34  (64%) | 8% | 46 (66%) | 35  (58%) | 8% | **197 (63%)** | **146**  **(56%)** | 7% |
| 6 to 10 | 17  (31%) | 12  (29%) | 2% | 23 (35%) | 19  (36%) | 1% | 16  (29%) | 23  (44%) | 15% | 17 (26%) | 18  (34%) | 2% | 20 (29%) | 19  (32%) | 3% | **93 (30%)** | **91 (35%)** | 5% |
| more than 10 | 1  (2%) | 3  (7%) | 5% | 5  (8%) | 4  (8%) | 0% | 10  (18%) | 7  (13%) | 5% | 1  (2%) | 1  (2%) | 0% | 4  (6%) | 5  (8%) | 2% | **21**  **(7%)** | **20**  **(8%)** | 1% |
| **Front-end flush type** |  |  |  |  |  |  |  |  |  |  |  |  |  |  |  |  |  |  |
| Cistern Flush | 53  (96%) | 38 (93%) | 3% | 38 (58%) | 33 (62%) | 4% | 43  (77%) | 38  (73%) | 4% | 64 (98%) | 50 (94%) | 4% | 61 (87%) | 54 (90%) | 3% | **260 (83%)** | **213 (82%)** | 1% |
| Pour flush | 0  (0%) | 2  (5%) | 5% | 23 (35%) | 18 (34%) | 1% | 10  (18%) | 7  (13%) | 5% | 0  (0%) | 0  (0%) |  | 6  (9%) | 2  (3%) | 7% | **39 (13%)** | **29 (11%)** | 2% |
| Hole (non-flush) | 1  (2%) | 1  (2%) | 0% | 4 (6%) | 1 (2%) | 4% | 3  (5%) | 6  (12%) | 7% | 1  (2%) | 0  (0%) |  | 3  (4%) | 4  (7%) | 3% | **12**  **(4%)** | **12 (5%)** | 1% |
| **Latrine floor type** |  |  |  |  |  |  |  |  |  |  |  |  |  |  |  |  |  |  |
| Washable floor | 52  (95%) | 39 (95%) | 0  (0%) | 61  (94%) | 47 (89%) | 5% | 50  (89%) | 45  (87%) | 2% | 63  (97%) | 53 (100%) | 3% | 61  (87%) | 53 (88%) | 1% | **287**  **(92%)** | **235 (91%)** | 1% |
| Non-washable floor | 3  (5%) | 2  (5%) | 0  (0%) | 4  (6%) | 5  (9%) | 3% | 6  (11%) | 6  (12%) | 1% | 2  (3%) | 0  (0%) | 3% | 9  (13%) | 7 (12%) | 1% | **24**  **(8%)** | **20 (8%)** | 0% |
| **Overall latrine front-end broken** |  |  |  |  |  |  |  |  |  |  |  |  |  |  |  |  |  |  |
| Yes | 22  (40%) | 15 (37%) | 3% | 26 (40%) | 7 (13%) | 27% | 30  (54%) | 22  (42%) | 12% | 23 (35%) | 9 (17%) | 18% | 20 (29%) | 16 (27%) | 2% | **121 (39%)** | **69 (27%)** | 12% |
| No | 33  (60%) | 25 (61%) | 8% | 39 (60%) | 42 (79%) | 3% | 26  (46%) | 29  (56%) | 10% | 42 (65%) | 43 (81%) | 1% | 50 (71%) | 44 (73%) | 2% | **190 (60%)** | **183 (71%)** | 11% |
| **Observation of faeces in or around latrine^2^** |  |  |  |  |  |  |  |  |  |  |  |  |  |  |  |  |  |  |
| Yes | 2  (4%) | 0  (0%) | 4% | 2  (3%) | 1  (2%) | 1% | 7  (13%) | 2  (4%) | 9% | 1  (2%) | 4  (8%) | 6% | 1  (1%) | 2  (3%) | 2% | **13**  **(4%)** | **9**  **(3%)** | 1 % |
| No | 53  (96%) | 40 (98%) | 2% | 62 (95%) | 47  (89%) | 6% | 49  (88%) | 49  (94%) | 6% | 64 (98%) | 49 (92%) | 6% | 69  (99%) | 58 (97%) | 2% | **297 (95%)** | **243 (94%)** | 1 % |
| **Visible moisture on latrine floor ^3^** |  |  |  |  |  |  |  |  |  |  |  |  |  |  |  |  |  |  |
| Yes | 18  (33%) | 19 (46%) | 13% | 35 (54%) | 23 (43%) | 11% | 31  (55%) | 32  (62%) | 7% | 30 (46%) | 19 (36%) | 10% | 16 (23%) | 22 (37%) | 14% | **130**  **(42%)** | **115 (44%)** | 3 % |
| No | 15  (27%) | 2  (5%) | 22% | 11 (17%) | 1  (2%) | 15% | 18  (32%) | 6  (12%) | 20% | 18 (28%) | 16 (30%) | 2% | 29 (41%) | 8 (13%) | 28% | **91 (29%)** | **33 (13%)** | 16% |
| Not differentiated | 22  (40%) | 10 (24%) | 16% | 18 (28%) | 11 (21%) | 7% | 7  (13%) | 7  (13%) | 0% | 17 (26%) | 13 (25%) | 1% | 25 (36%) | 22 (37%) | 1% | **89 (29%)** | **63 (24%)** | 5% |
| **Visible dirt on latrine floor ^3^** |  |  |  |  |  |  |  |  |  |  |  |  |  |  |  |  |  |  |
| Yes | 26  (47%) | 12 (29%) | 18% | 36 (55%) | 20 (38%) | 17% | 30  (54%) | 25  (48%) | 6% | 31 (48%) | 25 (47%) | 6% | 23 (33%) | 24 (40%) | 7% | **146 (47%)** | **95 (37%)** | 10% |
| No | 15  (27%) | 17 (41%) | 14% | 20  (31%) | 14 (26%) | 5% | 21  (38%) | 15  (29%) | 9% | 23 (35%) | 0 |  | 39 (56%) | 17 (28%) | 28% | **118 (38%)** | **88 (34%)** | 4% |
| Not differentiated | 14  (25%) | 2  (5%) | 20% | 8  (12%) | 1  (2%) | 10% | 5  (9%) | 5  (10%) | 5% | 11  (17%) | 14 (26%) | 9% | 8  (11%) | 11 (18%) | 7% | **46 (15%)** | **28 (11%)** | 4% |
| **Latrine used as storage ^3^** |  |  |  |  |  |  |  |  |  |  |  |  |  |  |  |  |  |  |
| Yes | 7  (13%) | 4  (10%) | 3% | 12  (18%) | 6  (11%) | 7% | 5  (9%) | 11  (21%) | 12% | 14 (22%) | 10  (19%) | 3% | 5  (7%) | 6  (10%) | 3% | **43 (14%)** | **37**  **(14%)** | 0 |
| No | 48  (87%) | 27  (66%) | 21% | 52 (80%) | 29  (55%) | 25% | 51  (91%) | 35  (67%) | 24% | 51 (78%) | 38  (72%) | 6% | 65 (93%) | 46  (77%) | 16% | **267**  **(86%)** | **175 (68%)** | 18% |
| **Observation of anal cleansing materials inside latrine ^4^** |  |  |  |  |  |  |  |  |  |  |  |  |  |  |  |  |  |  |
| Yes | 48  (87%) | 39  (95%) | 8% | 55  (85%) | 47  (89%) | 4% | 45  (80%) | 51  (98%) | 18% | 63  (97%) | 48  (91%) | 6% | 63  (90%) | 58  (97%) | 7% | **274**  **(88%)** | **243**  **(94%)** |  |
| No | 7  (13%) | 1  (2%) | 11% | 10  (15%) | 5  (9%) | 6% | 11  (20%) | 0  (0%) | 20% | 2  (3%) | 5  (9%) | 6% | 7  (10%) | 2  (3%) | 7% | **37**  **(12%)** | **13**  **(5%)** |  |
| **Type of anal cleansing material used reported by households ^5^** |  |  |  |  |  |  |  |  |  |  |  |  |  |  |  |  |  |  |
| Toilet paper | 29  (53%) | 27  (66%) | 13% | 16  (25%) | 30  (57%) |  | 38  (68%) | 29  (56%) | 12% | 28  (43%) | 30  (57%) | 14% | 57  (81%) | 50  (83%) | 2% | **168**  **(54%)** | **166**  **(64%)** | 10% |
| Newspaper | 0  (0%) | 4  (10%) | 10% | 1  (2%) | 4  (8%) | 6% | 7  (13%) | 1  (2%) | 11% | 0 | 0 | 0 | 1  (1%) | 0 | 1% | **9**  **(3%)** | **9**  **(3%)** | 0 |
| Toilet paper and newspaper | 24  (44%) | 5  (12%) | 32% | 24  (37%) | 4  (8%) | 29% | 11  (20%) | 12  (23%) | 3% | 37  (57%) | 12  (23%) | 34% | 9  (13%) | 6  (10%) | 3% | **105**  **(34%)** | **39**  **(15%)** | 19% |
| Toilet paper and water | 0  (0%) | 3  (7%) | 7% | 23  (35%) | 9  (17%) | 18% | 0  (0%) | 1  (2%) | 2% | 0  (0%) | 4  (8%) | 8% | 3  (4%) | 3  (5%) | 1% | **26**  **(8%)** | **20**  **(8%)** | 0% |
| Newspaper and water | 0  (0%) | 0  (0%) | 0  (0%) | 0  (0%) | 2  (4%) | 4% | 0  (0%) | 1  (2%) | 2% | 0  (0%) | 0  (0%) | 0% | 0  (0%) | 0  (0%) | 0% | **0**  **(0%)** | **3**  **(1%)** | 1% |
| Toilet paper, newspaper, and water | 0  (0%) | 2  (5%) | 5% | 0  (0%) | 3  (6%) | 6% | 0  (0%) | 7  (13%) | 13% | 0  (0%) | 6  (11%) | 11% | 0  (0%) | 0  (0%) | 0% | **0**  **(0%)** | **18**  **(7%)** | 7% |
| Water | 0  (0%) | 0  (0%) | 0  (0%) | 1  (2%) | 1  (2%) | 2% | 0  (0%) | 0  (0%) | 0% | 0  (0%) | 1  (2%) | 2% | 0  (0%) | 0  (0%) | 0% | **0**  **(0%)** | **2**  **(1%)** | 1% |
| **How do you dispose of your child faeces?** ^6, 7^ |  |  |  |  |  |  |  |  |  |  |  |  |  |  |  |  |  |  |
| In the toilet | 16  (29%) | 4  (10%) | 19% | 39  (60%) | 2  (4%) | 56% | 41  (73%) | 9  (17%) | 56% | 32  (49%) | 11  (21%) | 28% | 45  (64%) | 16  (27%) | 37% | **173**  **(56%)** | **42**  **(16%)** | 40% |
| Thrown or washed in the river | 0  (0%) | 0  (0%) | 0% | 4  (6%) | 0  (0%) | 6% | 1  (2%) | 0  (0%) | 2% | 2  (3%) | 0  (0%) | 3% | 0  (0%) | 0  (0%) | 0% | **7**  **(2%)** | **0**  **(0%)** | 2% |
| Thrown in bushes | 0  (0%) | 0  (0%) | 0% | 0  (0%) | 1  (2%) | 2% | 1  (2%) | 3  (6%) | 4% | 1  (2%) | 3  (6%) | 4% | 1  (1%) | 2  (3%) | 2% | **3**  **(1%)** | **9**  **(3%)** | 2% |
| Disposed together with solid waste | 12  (22%) | 7  (17%) | 5% | 5  (8%) | 13  (25%) | 17% | 4  (7%) | 13  (25%) | 18% | 8  (12%) | 7  (13%) | 1% | 9  (13%) | 21  (35%) | 22% | **38**  **(12%)** | **61**  **(24%)** | 12% |
| Buried in pit | 0  (0%) | 3  (7%) | 7% | 1  (2%) | 2  (4%) | 1% | 1  (2%) | 1  (2%) | 0% | 0  (0%) | 1  (2%) | 2% | 0  (0%) | 0  (0%) | 0% | **2**  **(1%)** | **7**  **(3%)** | 2% |
| Cleaned and burned to ignite firewood | 0  (0%) | 0  (0%) | 0% | 0  (0%) | 0  (0%) | 0% | 0  (0%) | 2  (4%) | 4% | 1  (2%) | 2  (4%) | 2% | 0  (0%) | 0  (0%) | 0% | **0**  **(0%)** | **4**  **(2%)** | 2% |
| **Observation of handwashing facilities inside latrine ^8^** |  |  |  |  |  |  |  |  |  |  |  |  |  |  |  |  |  |  |
| Yes | 5  (9%) | 1  (2%) | 7% | 9  (14%) | 3  (6%) | 8% | 1  (2%) | 4  (8%) | 6% | 5  (8%) | 3  (6%) | 2% | 4  (6%) | 5  (8%) | 2% | **24**  **(8%)** | **16 (6%)** | 2% |
| No | 49  (89%) | 38  (93%) | 4% | 56  (86%) | 49  (92%) | 6% | 55  (98%) | 46  (88%) | 10% | 60  (92%) | 50  (94%) | 2% | 66  (94%) | 55  (92%) | 2% | **286 (92%)** | **238 (92%)** | 0% |
| **Observation of handwashing facilities elsewhere in household ^9^** |  |  |  |  |  |  |  |  |  |  |  |  |  |  |  |  |  |  |
| Yes | 53  (96%) | 27  (66%) | 30% | 50  (77%) | 40  (75%) | 2% | 41  (73%) | 26  (50%) | 23% | 57  (88%) | 40  (75%) | 13% | 65  (93%) | 55  (92%) | 1% | **266 (86%)** | **188**  **(73%)** | 13% |
| No | 2  (4%) | 8  (20%) | 16% | 15  (23%) | 9  (17%) | 6% | 15  (27%) | 24  (46%) | 19% | 8  (12%) | 13  (25%) | 13% | 5  (7%) | 4  (7%) | 0% | **45 (14%)** | **58**  **(22%)** | 8% |
| **Type of handwashing facilities observed elsewhere in the household** |  |  |  |  |  |  |  |  |  |  |  |  |  |  |  |  |  |  |
| Tap with running water and sink | 35  (66%) | 17  (63%) | 3% | 28  (56%) | 29  (73%) | 17% | 7  (17%) | 6  (23%) | 6% | 39  (68%) | 26  (65%) | 3% | 35  (54%) | 43  (78%) | 24% | **144**  **(54%)** | **116**  **(64%)** | 10% |
| Bucket of water | 9  (17%) | 3  (11%) | 6% | 20  (40%) | 4  (10%) | 30% | 12  (29%) | 0  (0%) | 29% | 13  (23%) | 0  (0%) | 23% | 17  (26%) | 4  (7%) | 19% | **71 (27%)** | **11**  **(6%)** | 21% |
| Bucket with tap | 7  (13%) | 7  (26%) | 13% | 2  (4%) | 7  (18%) | 14% | 3  (7%) | 19  (73%) | 66% | 3  (5%) | 8  (20%) | 15% | 5  (8%) | 6  (11%) | 3% | **20**  **(8%)** | **47 (25%)** | 17% |
| Others | 2  (4%) | 0  (0%) | 4% | 0  (0%) | 0  (0%) | 0% | 19  (46%) | 1  (4%) | 42% | 2  (4%) | 6  (15%) | 11% | 8  (12%) | 2  (4%) | 8% | **31**  **(12%)** | **9**  **(5%)** | 7% |
| **Do you normally wash your hand after defecation? ^10^** |  |  |  |  |  |  |  |  |  |  |  |  |  |  |  |  |  |  |
| Yes | 51  (93%) | 40  (98%) | 5% | 63  (97%) | 52  (98%) | 1% | 55  (98%) | 51  (98%) | 0% | 63  (97%) | 52  (98%) | 1% | 70  (100%) | 58  (97%) | 3% | **302**  **(97%)** | **253**  **(98%)** | 1% |
| No | 4  (7%) | 1  (2%) | 5% | 2  (3%) | 1  (2%) | 1% | 1  (2%) | 0  (0%) | 2% | 2  (3%) | 0 | 3% | 0  (0%) | 1  (2%) | 2% | **9**  **(3%)** | **3**  **(1%)** | 2% |
| **Is soap used for handwashing?**^11^ |  |  |  |  |  |  |  |  |  |  |  |  |  |  |  |  |  |  |
| Always | 19  (35%) | 10  (24%) | 11% | 43  (66%) | 22  (42%) | 24% | 17  (30%) | 21  (40%) | 10% | 28  (43%) | 19  (36%) | 7% | 36  (51%) | 40  (67%) | 16% | **142 (46%)** | **112**  **(43%)** | 3% |
| Usually | 10  (18%) | 12  (29%) | 11% | 0  (0%) | 10  (19%) | 19% | 12  (21%) | 14  (27%) | 6% | 8  (12%) | 22  (42%) | 30% | 16  (23%) | 5  (17%) | 6% | **46 (15%)** | **63**  **(24%)** | 9% |
| Sometimes | 26  (47%) | 19  (46%) | 1% | 21  (32%) | 18  (34%) | 2% | 25  (45%) | 14  (27%) | 18% | 24  (37%) | 11  (21%) | 16% | 18  (26%) | 10  (17%) | 9% | **114 (37%)** | **72**  **(28%)** | 9% |
| Rarely | 0  (0%) | 1  (2%) | 2% | 1  (2%) | 2  (4%) | 2% | 2  (4%) | 0 | 4% | 5  (8%) | 0  (0%) | 8% | 0  (0%) | 0  (0%) | 0% | **8**  **(3%)** | **3**  **(1%)** | 2% |
| Never | 0  (0%) | 0  (0%) | 0  (0%) | 0  (0%) | 0  (0%) | 0  (0%) | 0  (0%) | 1  (2%) | 2% | 0  (0%) | 1  (2%) | 2% | 0  (0%) | 0  (0%) | 0% | **0**  **(0%)** | **2**  **(1%)** | 1% |
| **Walking outdoor without shoes – interviewee** ^12^ |  |  |  |  |  |  |  |  |  |  |  |  |  |  |  |  |  |  |
| Always | 0  (0%) | 10  (24%) | 24% | 5  (8%) | 8  (15%) | 7% | 10  (18%) | 14  (27%) | 9% | 7  (11%) | 26  (49%) | 38% | 13  (19%) | 18  (30%) | 11% | **35**  **(11%)** | **76**  **(29%)** | 18% |
| Usually | 5  (9%) | 2  (5%) | 4% | 14  (22%) | 3  (6%) | 16% | 10  (18%) | 4  (8%) | 10% | 17  (26%) | 9  (17%) | 9% | 12  (17%) | 6  (10%) | 7% | **58**  **(19%)** | **24**  **(9%)** | 10% |
| Sometimes | 12  (25%) | 20  (49%) | 24% | 24  (37%) | 25  (47%) | 10% | 31  (55%) | 20  (38%) | 17% | 32  (49%) | 11  (21%) | 28% | 29  (41%) | 8  (13%) | 28% | **130**  **(42%)** | **84**  **(32%)** | 10% |
| Rarely | 12  (22%) | 4  (10%) | 12% | 4  (17%) | 6  (11%) | 6% | 0  (0%) | 1  (2%) | 2% | 4  (6%) | 0  (0%) | 6% | 6  (9%) | 2  (3%) | 6% | **26**  **(8%)** | **13**  **(5%)** | 3% |
| Never | 24  (44%) | 5  (12%) | 32% | 18  (8%) | 10  (19%) | 11% | 5  (9%) | 12  (23%) | 14% | 5  (8%) | 7  (13%) | 5% | 10  (14%) | 25  (42%) | 28% | **62**  **(20%)** | **59**  **(23%)** | 3% |
| **Walking outdoor without shoes – other family members ^13^** |  |  |  |  |  |  |  |  |  |  |  |  |  |  |  |  |  |  |
| Always | 1  (2%) | 10  (24%) | 22% | 4  (6%) | 8  (15%) | 9% | 9  (16%) | 14  (27%) | 11% | 7  (11%) | 25  (47%) | 36% | 14  (20%) | 15  (25%) | 5% | **35**  **(11%)** | **72**  **(28%)** | 17% |
| Usually | 5  (9%) | 2  (5%) | 4% | 13  (20%) | 2  (4%) | 16% | 10  (18%) | 5  (10%) | 8% | 18  (28%) | 8  (15%) | 13% | 9  (13%) | 7  (12%) | 1% | **55**  **(18%)** | **24 (9%)** | 9% |
| Sometimes | 16  (20%) | 21  (51%) | 31% | 25  (38%) | 27  (51%) | 13% | 29  (52%) | 19  (37%) | 15% | 29  (45%) | 13  (25%) | 20% | 34  (49%) | 12  (20%) | 29% | **133 (43%)** | **92**  **(36%)** | 7% |
| Rarely | 11  (20%) | 3  (7%) | 13% | 4  (6%) | 6  (11%) | 5% | 3  (5%) | 0  (0%) | 5% | 5  (8%) | 1  (2%) | 6% | 2  (3%) | 1  (2%) | 1% | **25**  **(8%)** | **11**  **(4%)** | 4% |
| Never | 22  (40%) | 5  (12%) | 32% | 19  (29%) | 10  (19%) | 10% | 5  (9%) | 13  (25%) | 16% | 6  (9%) | 6  (11%) | 2% | 11  (16%) | 24  (40%) | 24% | **63**  **(20%)** | **58 (22%)** | 2% |
| **Reported diarrhoea in past month by households**^14^ |  |  |  |  |  |  |  |  |  |  |  |  |  |  |  |  |  |  |
| Yes | 4  (7%) | 8  (20%) | 13% | 10  (15%) | 6  (11%) | 4% | 17  (30%) | 10  (19%) | 11% | 8  (12%) | 7  (13%) | 1% | 9  (13%) | 8 (13%) | 0% | **48**  **(15%)** | **39**  **(15%)** | 0% |
| No | 51  (93%) | 33  (80%) | 13% | 55  (85%) | 46  (87%) | 2% | 39  (70%) | 41  (79%) | 9% | 57  (88%) | 46  (87%) | 1% | 61  (87%) | 49  (82%) | 5% | **263**  **(85%)** | **215**  **(83%)** | 2% |
| **Reported household members diagnosed with typhoid** ^15^ |  |  |  |  |  |  |  |  |  |  |  |  |  |  |  |  |  |  |
| Yes | 0  (0%) | 0  (0%) | 0% | 0  (0%) | 0  (0%) | 0% | 1  (2%) | 1  (2%) | 0% | 3  (5%) | 0  (0%) | 5 % | 2  (3%) | 1  (2%) | 1% | **6**  **(2%)** | **2**  **(1%)** | 1% |
| No | 55  (100%) | 41  (100%) | 0% | 65  (100%) | 52  (98%) | 2% | 55  (98%) | 51  (98%) | 0% | 62  (95%) | 53  (100%) | 5% | 68  (97%) | 57  (95%) | 2% | **305**  **(98%)** | **254**  **(98%)** | 0% |

^1^ two missing data in endline, ^2^ One missing data in baseline and seven in endline, ^3^one missing data in baseline and 46 in endline, ^4^three missing data in endline, ^5^two missing data in baseline and three in endline, ^6^ the sample size was smaller for child-faeces disposal as in baseline 82 households did not have any children at home and 95 households in endline, ^7^ 28 missing data in endline, ^8^one missing data in baseline and five in endline, ^9^ 13 missing data in endline, ^10^ three missing data in endline, ^11^ seven missing data in endline, ^12^ two missing data in endline, ^13^ five missing data in endline, ^14^ five missing data in endline , ^15^ three missing data in endline


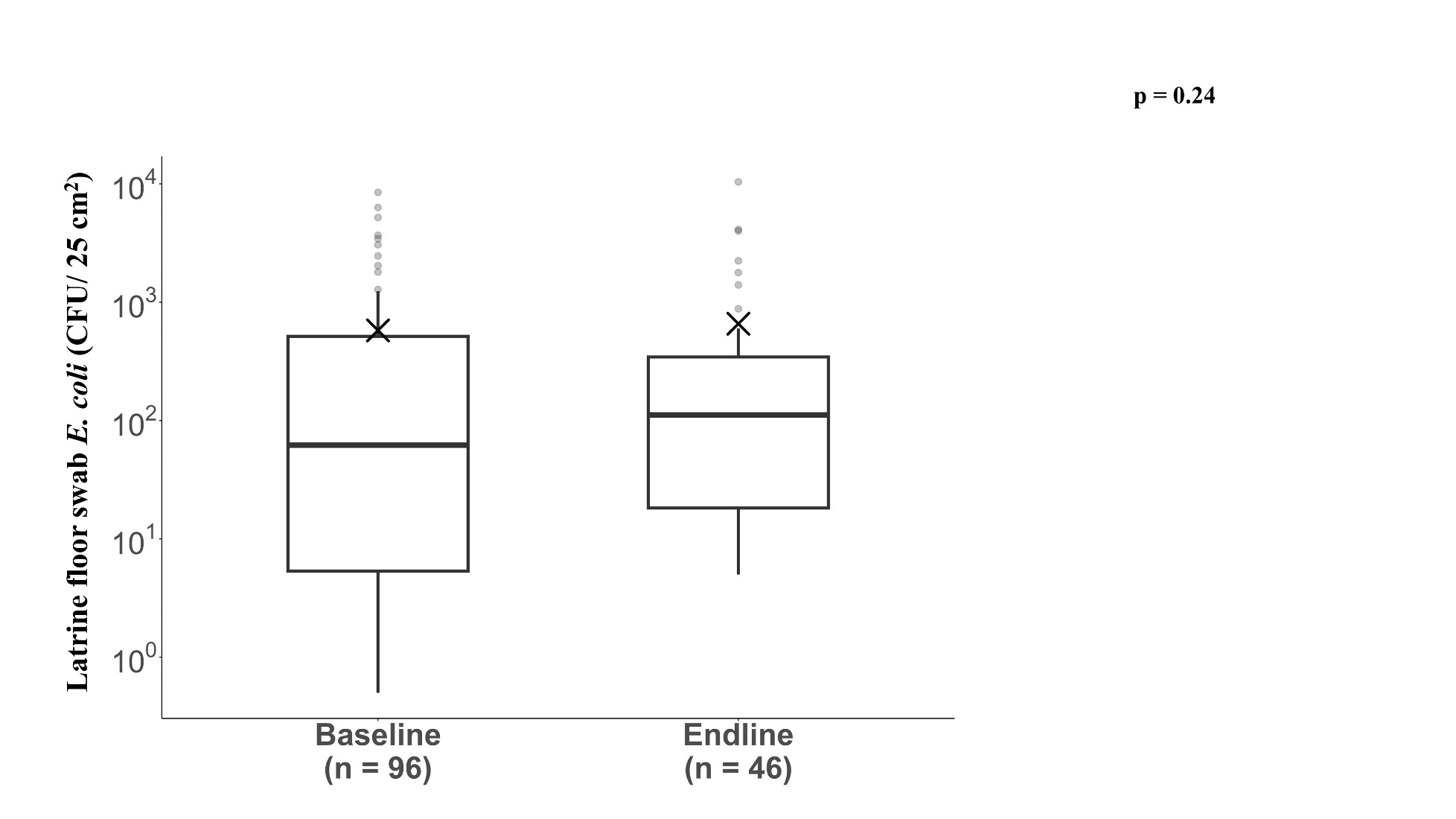


**Fig. S2** *E. coli* density (CFU/ 25 cm^2^) on the latrine floor from baseline (n = 96) and endline (n = 46). The box plot represents the median, quartiles, outlier (grey, round shape) and mean (×). The lower limit of detection is 1.0 CFU/25 cm^2^. Non-detects were substituted with half of the lower limit of detection values. The *E. coli* densities data presented are raw continuous data.

**Table S4:** *E. coli* density (CFU/25 cm^2^) on latrine floor with latrine front-end characteristics in baseline and endline sampling with a total of 142 latrines swab samples.

| **Description of categories** | **Categories** | **Sample size (n)** | **Mean *E. coli* (CFU/25 cm^2^) on latrine floor** | **p-value** |
| --- | --- | --- | --- | --- |
| **Household and latrine front-end characteristics** |  |  |  |  |
| Latrine floor type^1^ | Washable floor | 125 | 6.7 ×10^2^ | **0.05** |
|  | Non-washable floor | 17 | 1.3 ×10^2^ |  |
| Latrine front-end type ^2^ | Cistern flush | 102 | 5.6 ×10^2^ | 0.77 |
|  | Pour-flush | 28 | 8.4 ×10^2^ |  |
|  | Hole -type | 12 | 4.3 ×10^2^ |  |
| Latrine ownership^1^ | Private | 106 | 4.8 ×10^2^ | 0.463 |
|  | Shared | 35 | 9.9 ×10^2^ |  |
| Latrine location in reference to household ^1^ | Outside | 111 | 6.6 ×10^2^ | 0.82 |
|  | Inside | 30 | 4.2 ×10^2^ |  |
| No. of latrine users ^2^ | One to five users | 84 | 5.0 ×10^2^ | 0.81 |
|  | Six to ten users | 40 | 3.8 ×10^2^ |  |
|  | More than 10 users | 17 | 1.6 ×10^3^ |  |
| Reported diarrhoea in households in the past month^1^ |  |  |  |  |
|  | Yes | 20 | 8.7 ×10^2^ | 0.83 |
|  | No | 120 | 5.7 ×10^2^ |  |
| Highest education levels attended by household ^2^ | Primary level | 28 | 9.0 ×10^2^ | 0.07 |
|  | Secondary level | 85 | 5.3 ×10^2^ |  |
|  | Tertiary level | 27 | 5.4 ×10^2^ |  |
| **Latrine front-end maintenance** |  |  |  |  |
| Latrine front-end broken^1,3^ | Yes | 68 | 5.5 ×10^2^ | 0.60 |
|  | No | 72 | 6.7 ×10^2^ |  |
| Latrine flush broken^1, 4, 5^ | Yes | 22 | 5.0 ×10^2^ | 0.26 |
|  | No | 77 | 8.2 ×10^2^ |  |
| Observation faeces in or around latrine ^1, 3^ | Yes | 10 | 5.6 ×10^2^ | 0.25 |
|  | No | 130 | 6.2 ×10^2^ |  |
| Visual moisture on latrine floor^1, 5^ | Yes | 71 | 7.1 ×10^2^ | 0.14 |
|  | No | 34 | 4.0 ×10^2^ |  |
|  | Not differentiated | 25 | 5.1 ×10^2^ |  |
| Visual dirt on latrine floor^1,6^ | Yes | 86 | 6.6 ×10^2^ | 0.19 |
|  | No | 31 | 5.8 ×10^2^ |  |
|  | Not differentiated | 13 | 1.9 ×10^2^ |  |

^1^ Mann-Whitney U Test , ^2^ Kruskal-Wallis Test, ^3^ two missing data, ^4^within cistern flush latrines (n = 102), ^5^three missing data, ^6^12 missing data

**Table S5**: *E. coli* density (CFU/25 cm^2^) on various sampling locations in 12 latrines of in-depth front-end study with types of latrine front-end flush systems (cistern flush, pour-flush and hole).

| **Sampling locations** | **Sample number(n)** | **% of positive *E. coli* samples within each sampling location^2^** | **Number of non-detects in each sampling location** | **Overall mean *E. coli* within sampling location (CFU/25 cm^2^)** | **Overall *E. coli* range within each sampling location (CFU/25 cm^2^)** | **Cistern-flush latrines (n = 6)** | | | **Pour-flush latrines (n = 3)** | | | **Hole-type latrines (n = 3)** | | |
| --- | --- | --- | --- | --- | --- | --- | --- | --- | --- | --- | --- | --- | --- | --- |
|  |  |  |  |  |  | **n** | **mean** | **range** | **n** | **mean** | **range** | **n** | **mean** | **range** |
| Outside door | 12 | 0 | 12 | ND | ND | 6 | ND | ND | 3 | ND | ND | 3 | 0 | 0 |
| Outside lock^1^ | 5 | 20 | 1 | 1.2 | ND to 6.0 | 4 | 1.5 | ND to 6.0 | 1 | ND | ND | NA | NA | NA |
| Inside door | 12 | 8 | 11 | 4.3 | ND to 50.0 | 6 | ND | ND | 3 | 16.7 | ND to 50.0 | 3 | 0 | 0 |
| Inside lock^1^ | 5 | 0 | 5 | ND | ND | 4 | ND | ND | 1 | ND | ND | NA | NA | NA |
| Latrine floor | 12 | 83 | 2 | 1.0 × 10^3^ | ND to 6.0 × 10^3^ | 6 | 94.4 | ND to 4.2 × 10^2^ | 3 | 3.8 × 10^3^ | 2.1 × 10^2^ to 6.0 × 10^3^ | 3 | 91.3 | 36.0 to 1.8 × 10^2^ |
| Latrine cover | 9 | 44 | 5 | 7.8 | ND to 52.5 | 6 | 11.6 | ND to 52.5 | 2 | ND | ND | 1 | ND | ND |
| Latrine seat | 12 | 58 | 5 | 60.0 | ND to 5.6 × 10^2^ | 6 | 97.5 | ND to 5.6 × 10^2^ | 3 | 18.5 | 0 to 55.6 | 3 | 26.0 | 15.0 to 37.0 |
| Flush button | 6 | 33 | 4 | 0.6 | ND to 2.0 | 6 | 0.6 | ND to 2.0 | NA | NA | NA | NA | NA | NA |
| Mid wall | 12 | 75 | 3 | 7.8 | ND to 22.0 | 6 | 10 | ND to 22.0 | 3 | 9.7 | 3.0 to 20.0 | 3 | 1.4 | ND to 4.0 |
| Lower wall | 12 | 17 | 10 | 4.0 | ND to 35.0 | 6 | 8.0 | ND to 35.0 | 3 | ND | ND | 3 | ND | ND |
| **Total** | **97** |  |  |  |  |  |  |  |  |  |  |  |  |  |

^1^ outside and inside lock expressed as CFU per swab, ^2^percentage calculated with total sample number for each surface (n), NA = Not applicable, ND = Not detected


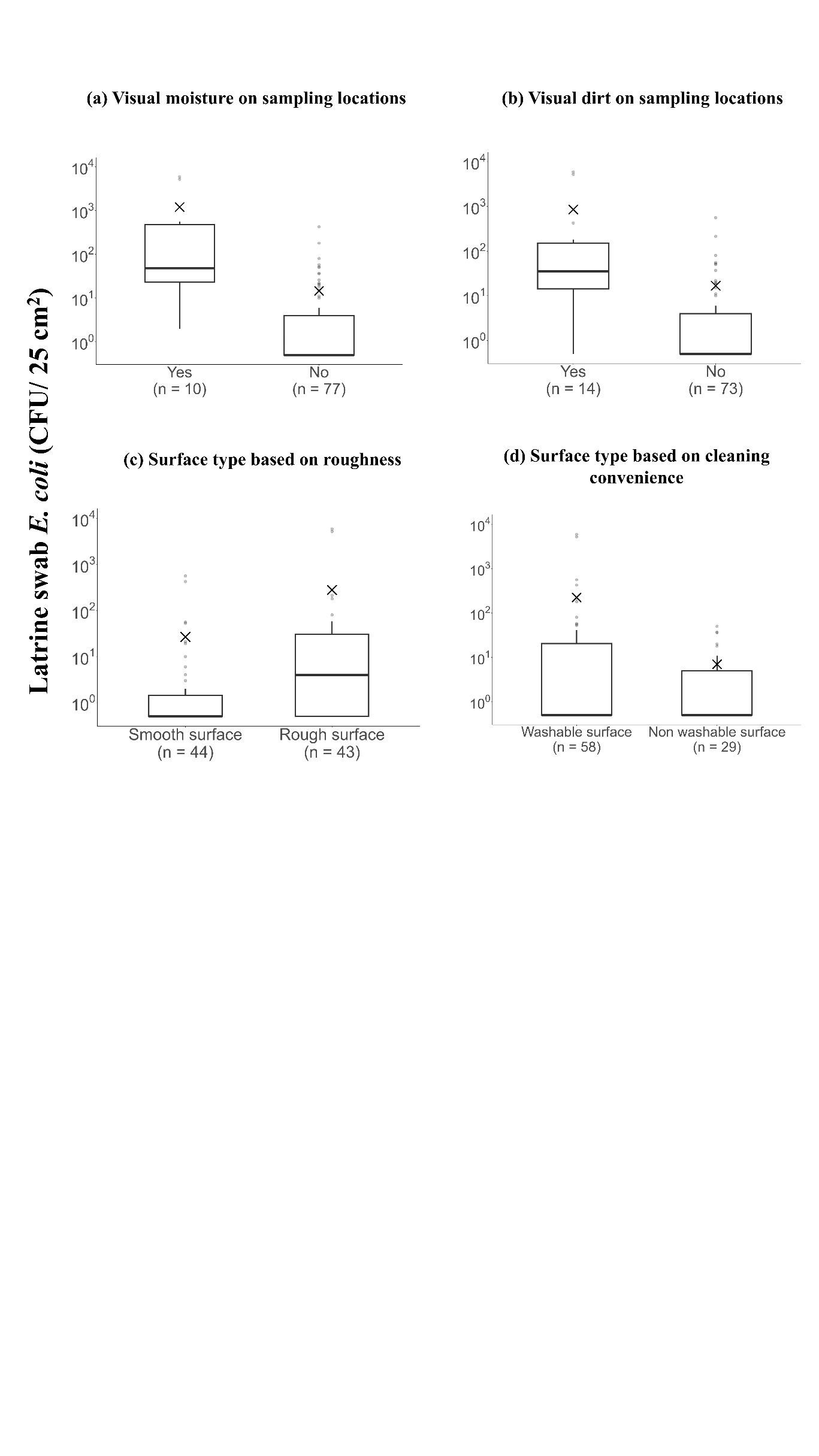


**Fig. S3** *E. coli* density (CFU/ 25 cm^2^) on overall sampling surfaces (excluding the inside and outside lock) in 12 latrines of in-depth front-end study with latrine front-end maintenance (87 surfaces, excluding outside and inside lock). The box plot represents the median, quartiles, outlier (grey dots) and mean (×). a) *E. coli* and visual presence of moisture observed on the surfaces during sampling; b) *E. coli* and visual presence of dirt observed on the surfaces during sampling; c) *E. coli* and type of surface based on visual surface roughness; d) *E. coli* and type of surface based on cleaning convenience. The lower limit of detection is 1.0 CFU/25 cm^2^. Non-detects were substituted with half of the lower limit of detection value. The *E. coli* densities data presented are raw continuous data.


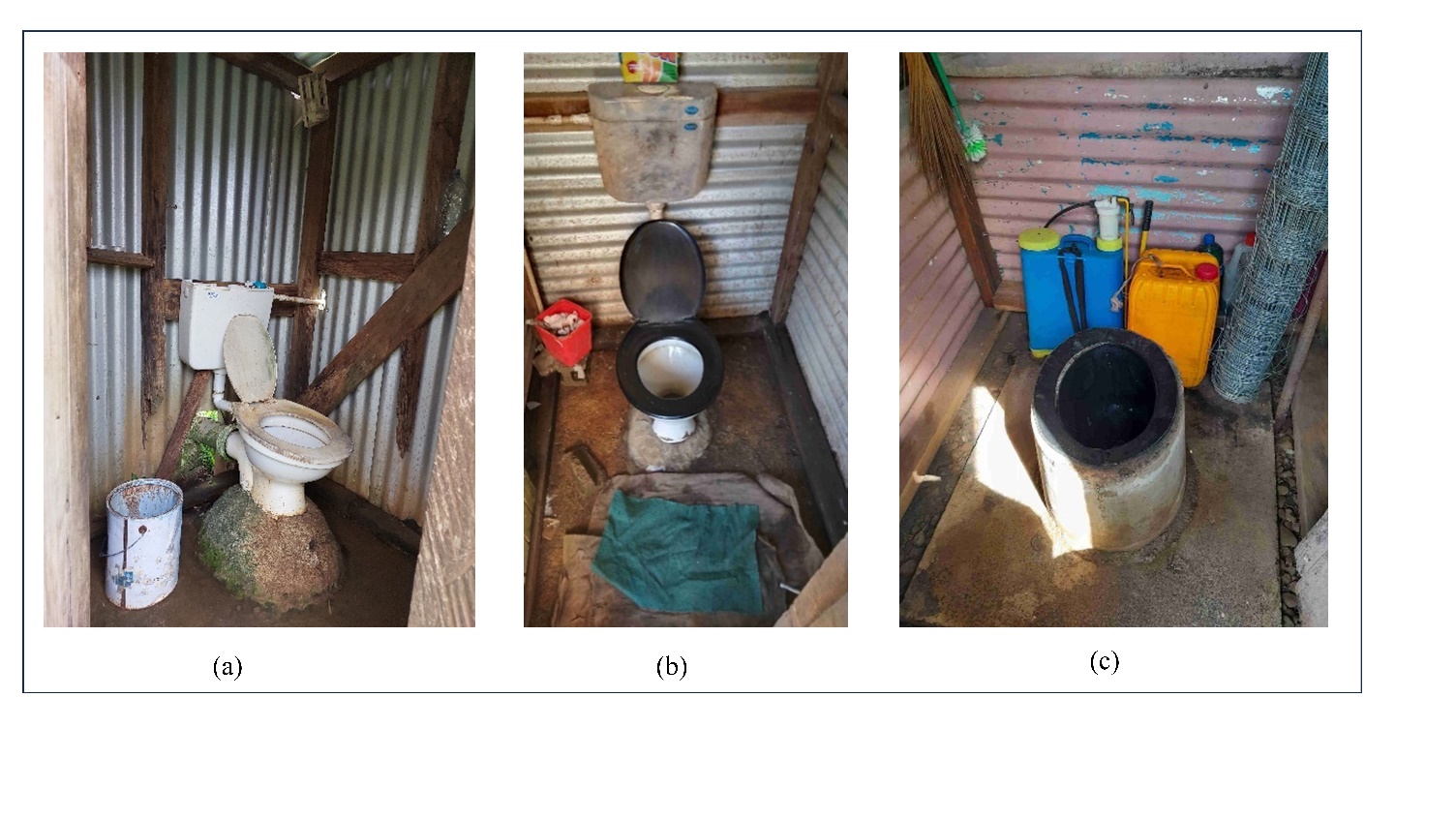


**Fig. S4** Latrine usage behaviour observed in rural Fiji: (a) Cistern flush latrine used as pour-flush after the cistern is broken; (b) Cistern flush latrine with the washable floor made of course concrete with moist floor and toilet mat; c) Latrine front-end used as storage for agricultural tools and jerry cans
